# *Akkermansia muciniphila* protects diabetes-associated cognitive decline by enhancing leucine catabolism and suppressing neuronal excessive mitophagy

**DOI:** 10.1080/19490976.2026.2739666

**Published:** 2026-09-30

**Authors:** Xianxi Ye, Qingpeng Cai, Yulong Li, Mingjie You, Junli Chen, Yangjie Pan, Ming Zhu, Zhuofan Lu, Siyuan Chen, Yan Lin, Weiru Zhang, Xuelian Liu, Zhen Wang, Guangliang Hong, Wenjun Wu, Hong Zheng

**Affiliations:** a State Key Laboratory of Macromolecular Drugs and Large-scale Preparation, School of Pharmaceutical Sciences, Wenzhou Medical University, Wenzhou, China; b Forensic Medicine Center, Wenzhou Medical University, Wenzhou, China; c Department of Endocrinology and Metabolism, The First Affiliated Hospital of Wenzhou Medical University, Wenzhou, China; d Department of Neurology, The First Affiliated Hospital of Wenzhou Medical University, Wenzhou, China; e Department of Emergency Medicine, The First Affiliated Hospital of Wenzhou Medical University, Wenzhou, China; f Department of Emergency Medicine, The Quzhou Affiliated Hospital of Wenzhou Medical University, Quzhou People’s Hospital, Quzhou, China

**Keywords:** *Akkermansia*, cognition, diabetes, leucine, gut–brain axis, mitophagy

## Abstract

Dysregulation of the gut–brain metabolic axis has been implicated in the progression of diabetes-associated cognitive decline (DACD), yet its underlying mechanism remains largely unknown. Herein, we identified a low abundance of *Akkermansia muciniphila* (AKK) and high leucine levels as key driving factors of DACD in both diabetic mice and patients. Our results revealed that AKK bacteria hold the capacity to catabolize leucine, and the elevated leucine levels observed in diabetic mice and patients are partly attributed to a low abundance of AKK bacteria. Moreover, leucine supplementation and deprivation further confirmed that excessive leucine accelerated and aggravated the progression of DACD in mice. Mechanistically, leucine directly modulated the phosphorylation of TBK1 and, in turn, phosphorylated the autophagy adaptor OPTN, thereby enhancing its binding capacity to LC3 and Ub chains and triggering neuronal excessive mitophagy. Therefore, this study uncovers a new mechanism of the gut-brain metabolic axis involved in DACD progression and provides a novel perspective for gut-targeted therapy of cognitive disorders.

## Graphical abstract

**Figure:**
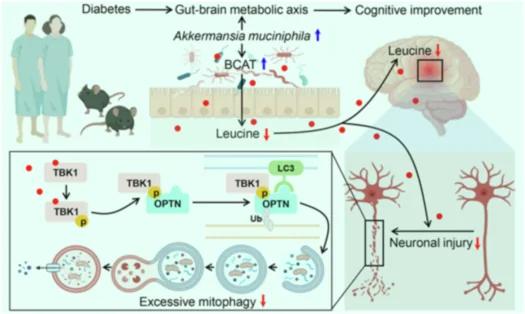

## 1. Introduction

Diabetes comprises a spectrum of chronic metabolic disorders due to insulin deficiency or insulin resistance and characterized by hyperglycemia.^1^ Diabetes causes complications involving many organ systems, resulting in a lower quality of life and increased risk of mortality.^2^ Among these complications, diabetes-associated cognitive decline (DACD) has received growing attention and is increasingly recognized as a risk factor of dementia.^3^ Several potential pathogenic mechanisms of DACD have been reported, including metabolic disorder,^4^ neuroinflammation,^5^ oxidative injury,^6^ mitochondrial dysfunction,^7^ and ferroptosis.^8^ Yet, the pathophysiological mechanisms of DACD remain largely unknown, posing challenges to its clinical diagnosis and treatment.

In recent years, scientists have begun exploring the pathogenic causes of DACD from the gut microbiome and its metabolites.^9^ The gut microbiota can modulate behavior and brain function through diverse pathways, a concept known as the gut-brain axis.^10^ ^,^ ^11^ Notably, microbial metabolites may serve as key mediators of the gut‒brain axis in DACD. For example, acetate depletion was shown to suppress hippocampal synaptophysin via vagus nerve inhibition, thereby accelerating cognitive impairment in T1D mice.^12^ The microbiota-derived glutamate was found to decrease microglial activation and proinflammatory factors and then relieve DACD in mice.^13^ In addition, fecal microbiota transplantation from healthy mice alleviated the development of DACD by increasing aspartate levels from the gut to brain and suppressing NLRP3 inflammasome and neuroinflammation.^14^ At present, a wide range of microbial metabolites have been identified as signaling molecules involving the gut-brain communication, such as kynurenine metabolites,^15^ short-chain fatty acids,^16^ bile acids^17^ and indole derivatives.^18^ Thus, it can be seen that investigation on the microbial metabolism is a promising method to uncover the gut–brain metabolic axis mechanisms of DACD.

In the present study, we identified that a low abundance of *Akkermansia muciniphila* (AKK) and high leucine levels may serve as key driving factors of DACD in both diabetic mice and patients. *In vitro* fermentation and transplantation experiments confirmed that AKK bacteria possess the capacity to catabolize leucine. Then, leucine supplementation and deprivation were employed to further clarify the direct causal relationship between elevated leucine levels and DACD. Besides, using an integrated method of multi-omics analyses, target screening, and gene knockdown, we elucidated the direct molecular targets and key signaling pathways that mediate the deleterious effect of excessive leucine in DACD. Finally, we further validated the close association among AKK bacteria, leucine, and cognitive function in diabetic patients. This study attempts to elucidate the gut‒brain metabolic axis underlying DACD, thereby offering a new perspective and strategy for gut-targeted therapy of cognitive disorders.

## 2. Results

### 2.1. Gut microbiota mediates cognitive decline and neuronal injury in T1D mice

In the present study, we first constructed a T1D mouse model via STZ administration. Cognitive performance, including spatial learning and memory, was then examined at the 11th week using the Morris water maze (MWM) assessment (Figure S1a). During the four consecutive days of training, T1D mice required a notably longer time to reach the hidden platform than normal control (CON) mice (Figure S1b, *p* = 0.02), indicating that T1D caused a deficit in learning capacity. The swimming trajectories of CON and T1D mice were illustrated in Figure S1c, from which several cognitive indicators were calculated during the test period. We found that T1D mice had lower memory ability compared with CON mice, as indicated by fewer platform crossings (Figure S1d) and reduced proportions of both swimming time (Figure S1e) and swimming distance (Figure S1f) within the target platform quadrant. These results indicate that T1D mice developed DACD in the later stages of disease progression.

Although STZ has been widely used to induce animal models of diabetes,^19^ we further investigated whether STZ directly affects learning and memory abilities in mice during model induction. Given that STZ exerts its cytotoxicity through cellular uptake via GLUT2,^20^ we first examined GLUT2 expression in the pancreas and hippocampus. The results show that GLUT2 was highly expressed in pancreatic islet cells but showed a relatively low expression in neuronal cells within the hippocampal CA1, CA3, and DG regions (Figures S2a, S2b). Then, we determined the time-course profile of STZ concentrations after intraperitoneal injection (Figure S2c), and found that the concentration of STZ in the serum and pancreas peaked at approximately 5 min and became undetectable by 30 min (Figures S2d, S2e), indicating rapid clearance. Notably, STZ was not detected in the hippocampus at any examined time point after intraperitoneal injection (Figure S2e). *In vitro* exposure experiments revealed that treatment with 10  mM STZ for 2 h significantly reduced SH-SY5Y cell viability (Figure S2f), but such an exposure condition is unlikely to occur during the induction of diabetic mouse models by intraperitoneal STZ injection. Finally, a T1D mouse model was established by intraperitoneal injection of STZ for five consecutive days, and learning and memory abilities were assessed by the MWM test after 4 weeks (Figure S2g). No significant differences were observed between T1D and CON mice in the escape latency (Figure S2h), the proportions of swimming time (Figure S2i) and swimming distance (Figure S2j) in the original platform region, as well as platform frequency (Figure S2k). Therefore, our results demonstrate that the intraperitoneal STZ administration used to induce diabetes in the mouse models in this study did not directly affect hippocampal neurons and cognitive functions of the mice.

To determine whether the gut microbiota directly contributes to DACD, the fecal microbiota collected from the CON mice were transferred into the T1D mice, followed by assessment of cognitive performance using the MWM test (Figure 1a). FMT significantly shortened the escape latency of T1D mice to a level comparable to that of CON mice (Figure 1b, *p* = 0.002), indicating that FMT improved the learning ability of T1D mice. The swimming trajectories of T1D mice that received FMT became more similar to those of CON mice (Figure 1c). Besides, we also observed that the platform frequency (Figure 1d) and the proportions of swimming time (Figure 1e) and swimming distance (Figure 1f) in the original platform region were notably increased in the T1D mice after FMT, and no significantly altered relative to CON mice. Thus it can be seen that T1D-induced memory impairment can be relieved after FMT from CON mice.

**Figure 1.**
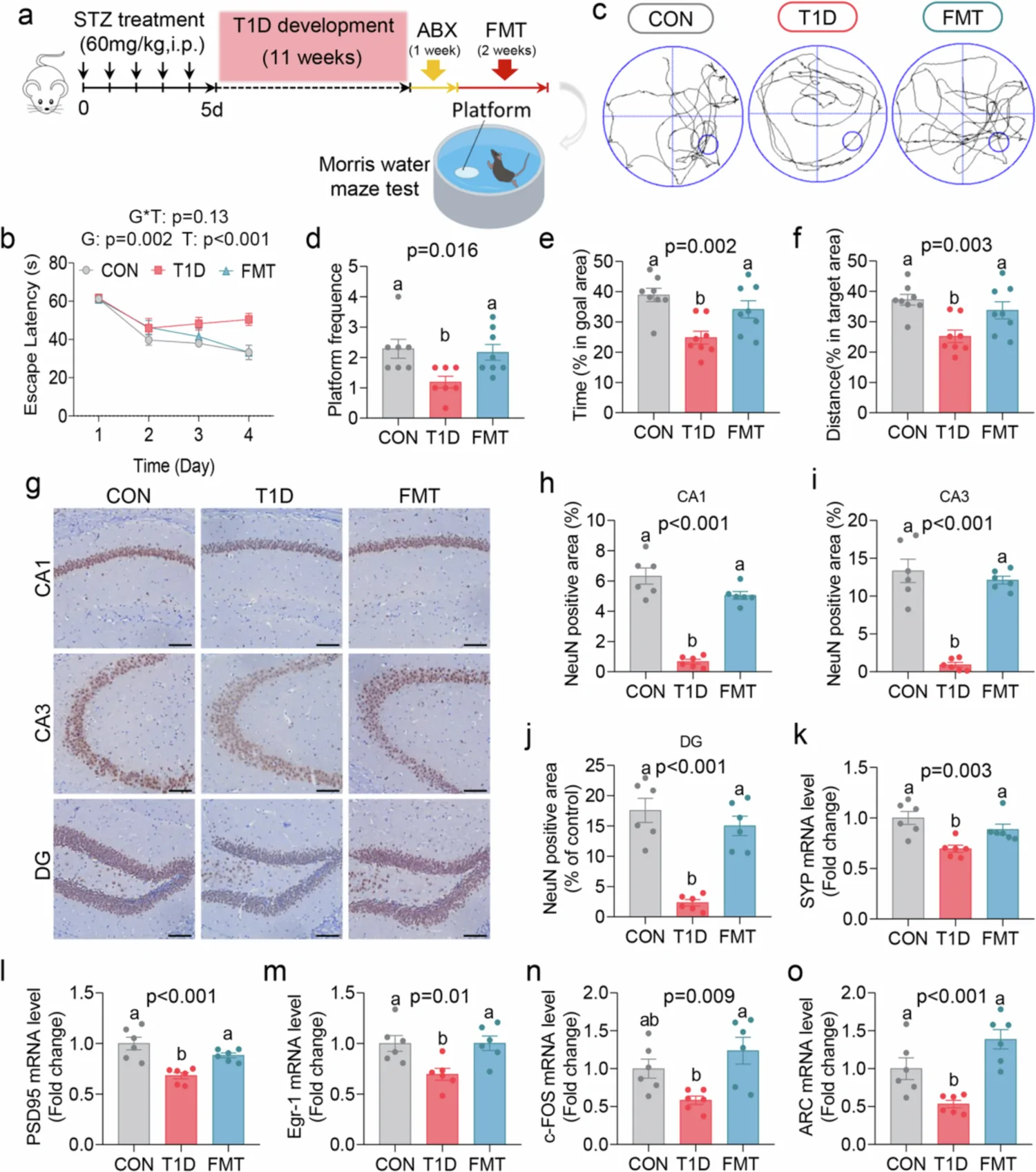
Transplantation of fecal microbiota from healthy mice alleviates neuronal injury and cognitive decline in diabetic mice. (a) Experimental procedure: In this study, type 1 diabetic (T1D) mice were established by intraperitoneal injection of streptozotocin (STZ). After 11 weeks of T1D progression, the mice were administered an antibiotic (ABX) cocktail for one week to eliminate the endogenous gut microbiota, followed by oral gavage transplantation of the fecal microbiota (FMT) from healthy donor mice for 2 weeks, and then learning and memory abilities were assessed using the Morris water maze (MWM) test. (b) The escape latency during a 4-day training period of the MWM test in normal control (CON) mice, T1D mice, and T1D mice receiving FMT from healthy donors (*n* = 7–8). (c) The swimming trajectories of CON, T1D, and FMT mice during the test period of the MWM test. (d–f) The platform frequency (d) and percentages of swimming time (e) and distance (f) in the original platform area in CON, T1D, and FMT mice during the test period of the MWM test (*n* = 7–8). (g–j) Representative NeuN staining images (g) and the corresponding quantitative data in the CA1 (h), CA3 (i), and DG (j) regions of the hippocampus in CON, T1D, and FMT mice (*n* = 6). Scale bar = 100 μm. (k–o) The mRNA expression levels of synaptic plasticity-related proteins, including SYP (k), PSD95 (l), EGR-1 (m), c-FOS (*n*), and ARC (o), in the hippocampus of CON, T1D, and FMT mice by RT-qPCR (*n* = 6). Data were presented as the means ±  standard errors. The statistical difference among different groups were analyzed by one-way ANOVA with a post-hoc Dunnett’s test for multiple comparisons, and different lowercase letters indicate statistically significant differences among groups (*p* < 0.05). The significant difference in the escape latency during a 4-day training period was evaluated by two-way repeated measure ANOVA: G*T, interaction effect of group and time; G, main effect of group; T, main effect of time.

Next, we performed histopathological staining to observe hippocampal neurons in different regions. Compared with those in CON mice, the H&E staining results illustrated that hippocampal neurons in the CA1, CA3, and DG regions exhibited disordered arrangement and nuclear shrinkage in T1D mice, which were markedly ameliorated after FMT (Figure S3a). The Nissl staining results revealed that the number of Nissl bodies was reduced in different hippocampal regions of T1D mice, whereas FMT markedly attenuated the loss of Nissl bodies in T1D mice (Figure S3b). Furthermore, compared with those of CON mice, significant reductions in the NeuN-positive areas were observed in the CA1 (Figure 1g, h), CA3 (Figure 1g, i), and DG (Figure 1g, j) regions of T1D mice, but the levels were notably elevated after FMT and showed no significant differences with CON mice. Finally, the gene expression levels of synaptic plasticity-related proteins in the hippocampus, and found that T1D mice had notably reduced levels of SYP (Figure 1k), PSD95 (Figure 1l), EGR-1 (Figure 1m), c-FOS (Figure 1n), and ARC (Figure 1o) relative to CON mice. These alterations were substantially alleviated after FMT. Our results imply that the gut microbiota plays a critical role in T1D-associated neuronal damage and cognitive impairment.

### 2.2. Gut microbiota shapes BCAAs metabolism in T1D mice

We employed 16S rRNA sequencing to analyze the alterations in the gut microbiota of T1D mice after FMT. The PCoA result illustrates clear separations in the microbial patterns among CON, T1D, and FMT mice (Figure 2a), indicating that FMT reshaped the gut microbiota in the T1D mice. At the phylum level, T1D mice showed a lower relative abundance of *Verrucomicrobia* and a higher abundance of *Firmicutes* than CON mice; of note, both alterations were restored in T1D mice that received FMT (Figure 2b). We then applied volcano plot analysis to screen for differentially abundant genera between T1D and CON mice (Figure 2c) and between T1D and FMT mice (Figure 2d). Subsequent Venn diagram analysis identified 16 gut microbial genera that were significantly changed in T1D mice compared with CON mice and that were also modulated by FMT (Figure 2e). These 16 gut microbes were listed as a heatmap in Figure 2f, where we observed that T1D mice had significantly higher abundances of *Weissella, Candidatus_Stoquefichus, Prevotellaceae_NK3B31_group, ASF356, Eubacterium_ventriosum, Eubacterium_brachy, Anaerofustis*, and *Alloprevotella* than CON mice, but their abundances were markedly decreased in T1D mice after FMT. In addition, the abundances of *Parasutterella, Parabacteroides*, and *Akkermansia* were significantly reduced in T1D mice compared with CON mice, whereas FMT substantially restored their levels (Figure 2f).

**Figure 2.**
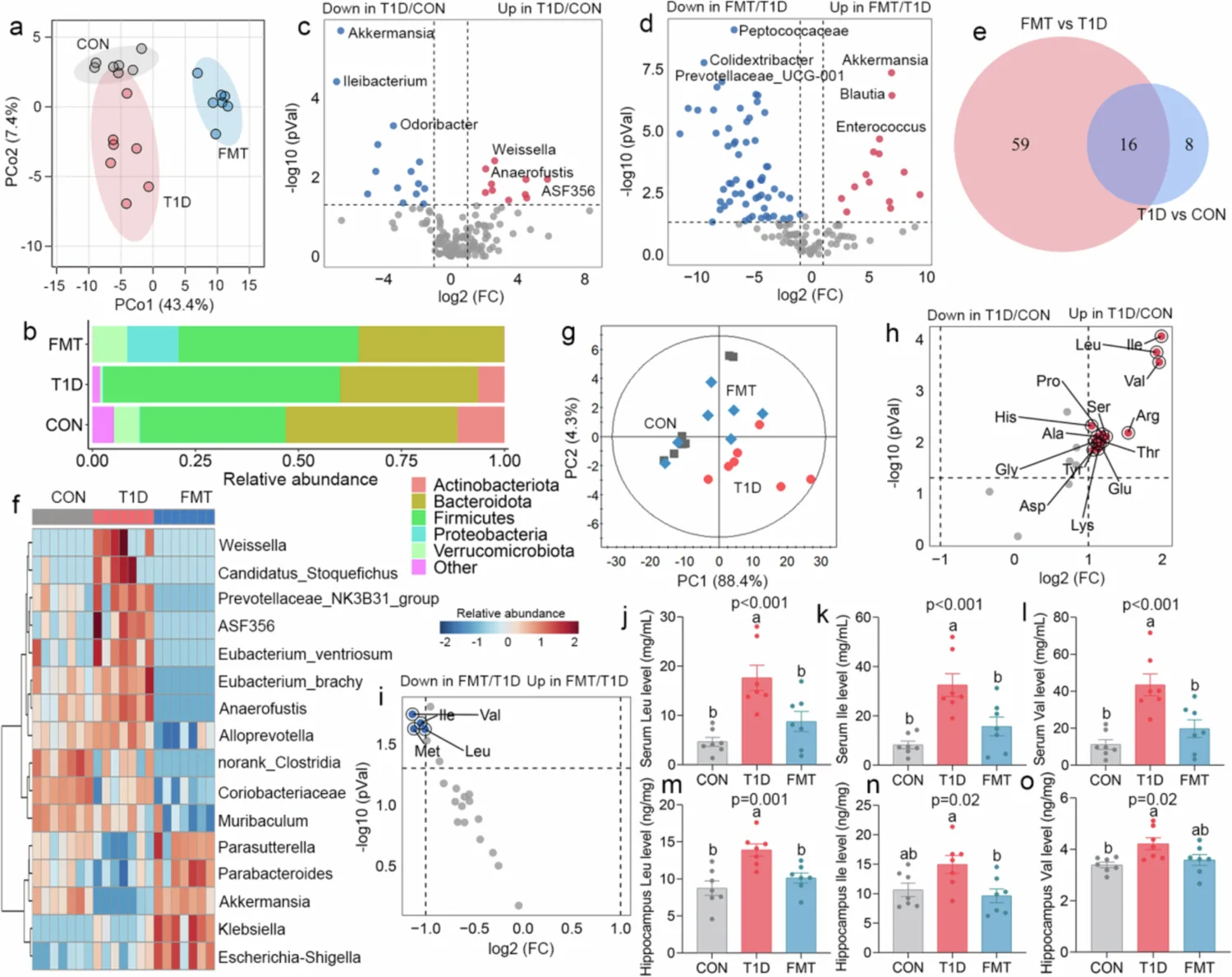
Transplantation of the fecal microbiota from healthy mice remodels BCAAs metabolism in diabetic mice. (a) PCoA results showing *β*-diversity of the gut microbiota in normal control (CON) mice, type 1 diabetic (T1D) mice, and T1D mice receiving FMT from healthy donors (*n* = 7). (b) The relative abundance of the gut microbiota at the phylum level in CON, T1D, and FMT mice was determined by 16S rRNA gene sequencing. (c, d) Significantly altered microbes were selected by the volcano plot analysis at the genus level between CON and T1D mice (c) as well as between T1D and FMT mice (d). (e) The Venn diagram analysis was used to identify key gut microbes that were markedly altered in T1D mice relative to CON mice but also varied after FMT. (f) Heatmap showing the changes of key gut microbes in CON, T1D and FMT mice (*n* = 7). (g) PCA classification based on amino acid profiles in CON, T1D, and FMT mice (*n* = 7). (h, i) Significantly altered amino acids were selected by the volcano plot analysis between CON and T1D mice (h) as well as between T1D and FMT mice (i). (j–l) The levels of leucine (j), isoleucine (k), and valine (l) in the serum of CON, T1D, and FMT mice by LC-MS analysis (*n* = 7). (m–o) The levels of leucine (m), isoleucine (*n*), and valine (o) in the hippocampus of CON, T1D, and FMT mice by LC-MS analysis (*n* = 7). Data were presented as the means ±  standard errors. The statistic difference among different groups was analyzed by one-way ANOVA with a post-hoc Dunnett’s test for multiple comparisons, and different lowercase letters indicate statistically significant differences among groups (*p* < 0.05).

To assess the effect of microbial alterations on host amino acid metabolism, we analyzed serum amino acid profiles in CON, T1D, and FMT mice using UPLC-MS. PCA analysis showed a distinct separation of T1D mice from the other two groups, whereas FMT-treated mice clustered more closely with CON mice (Figure 2g), indicating that FMT reversed amino acid metabolic disorders in T1D mice. Subsequently, the volcano plot analysis was performed to identify significantly altered amino acids between CON and T1D mice (Figure 2h) as well as between T1D and FMT mice (Figure 2i). Among these metabolites, branched-chain amino acids (BCAAs) showed the most pronounced and consistent alterations. Specifically, the serum levels of leucine (Figure 2j), isoleucine (Figure 2k), and valine (Figure 2l) were significantly elevated in T1D mice compared with those in CON mice, whereas FMT treatment markedly reduced their accumulation in T1D mice. Besides, we observed similar results in the hippocampus of mice (Figure 2m–o). Taken together, FMT from healthy mice can remodel BCAAs metabolism in T1D mice.

### 2.3. Leucine synergized with high glucose promotes cognitive decline in T1D mice

To investigate the impact of BCAAs on neuronal cells, SH-SY5Y cells were treated with different concentrations of leucine, isoleucine, or valine for 48 h *in vitro*, followed by cell viability determination. We found that SH-SY5Y cell viability was significantly reduced as the concentration of leucine above 25 mM (Figure S4a) under normal glucose (NG, 5.5 mM) conditions. However, within the same concentration range, treatment with valine (Figure S4b) or isoleucine (Figure S4c) showed no significant effect on SH-SY5Y cell viability under a NG condition. These findings indicate that, compared with valine and isoleucine, excessive leucine had a more pronounced adverse impact on neuronal viability. Interestingly, we further observed that leucine at a concentration of 10 mM exerted a detrimental effect on SH-SY5Y cell viability under a high-glucose (HG) condition (Figure S4d), implying that excessive leucine and HG exhibited a synergistic effect on the impairment of neuronal viability. Moreover, we found that the combined treatment with leucine and HG induced adverse effects on SH-SY5Y cell viability as early as 24 h (Figure S5). This result further demonstrated the synergistic cytotoxicity between leucine and HG.

To explore the influence of leucine on neuronal and cognitive functions in diabetic mice, we administered exogenous leucine supplementation to T1D mice for 8 (Figure 3a) and 12 weeks (Figure S6a). After 8 weeks, leucine-treated T1D (T1DL) mice exhibited a longer escape latency than both CON and T1D mice (Figure 3b, *p* = 0.04). Representative swimming paths during the MWM test are shown in Figure 3c. Compared with CON and T1D mice, T1DL mice spent markedly less of their movement trajectory within the original platform region, suggesting poorer spatial memory ability. Moreover, the platform frequency (Figure 3d) and the proportions of swimming time (Figure 3e) and swimming distance (Figure 3f) within the original platform region were not notably altered between T1D and CON mice at 8 weeks, but these indicators were drastically reduced in T1DL mice. Our findings indicate that excessive leucine accelerated the progression of T1D-induced cognitive decline in mice. Subsequently, we also observed from the H&E staining that neuronal cells were in a disordered arrangement and nuclear shrinkage in the hippocampal CA1, CA3, and DG regions of T1DL mice relative to T1D mice (Figure S7a). According to both Nissl and NeuN staining, T1DL mice exhibited significantly lower levels of Nissl bodies (Figure S7b) and NeuN-positive areas (Figure 3g–j) in the hippocampal regions than T1D mice, suggesting that excessive leucine can induce hippocampal neuronal damage in T1D mice. Compared with T1D mice, moreover, T1DL mice showed markedly decreased hippocampal mRNA expression of synaptic plasticity-associated markers, including SYP, PSD95, EGR-1, c-FOS, and ARC (Figure 3k).

**Figure 3.**
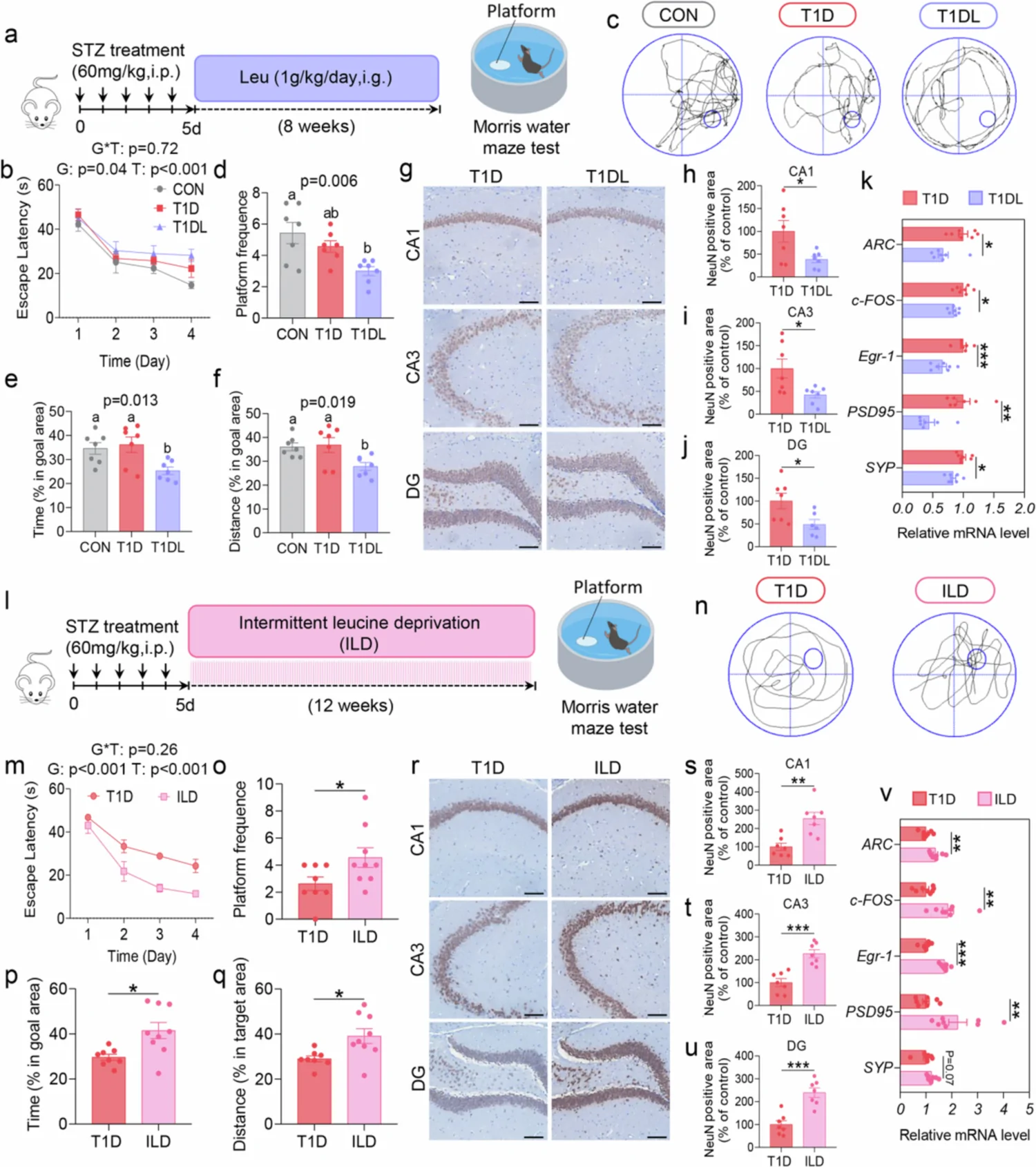
Excessive leucine accelerates neuronal injury and cognitive decline in diabetic mice. (a) Experimental procedure: In this study, type 1 diabetic (T1D) mice were established by intraperitoneal injection of streptozotocin (STZ) and treated with leucine for 8 weeks. Subsequently, the learning and memory abilities of the mice were evaluated by using the Morris water maze (MWM) test. (b) The escape latency during a 4-day training period of the MWM test in normal control (CON) mice, T1D mice, and T1D mice treated with leucine for 8 weeks (T1DL) (*n* = 7). (c) The swimming trajectories of CON, T1D, and T1DL mice during the test period of the MWM test. (d–f) The platform frequency (d) and the percentages of swimming time (e) and distance (f) in the original platform area in CON, T1D, and T1DL mice during the test period of the MWM test (*n* = 7). (g–j) Representative NeuN staining images (g) and the corresponding quantitative data in the CA1 (h), CA3 (i), and DG (j) regions of the hippocampus in CON, T1D, and T1DL mice (*n* = 7). Scale bar = 100 μm. (k) The mRNA expression levels of synaptic plasticity-related proteins in the hippocampus of CON, T1D, and T1DL mice by RT-qPCR (*n* = 7). (l) Experimental procedure: T1D mice were fed a leucine-deficient diet every other day for 12 weeks, and then their learning and memory abilities were assessed by using the Morris water maze (MWM) test. (m) The escape latency during a 4-day training period of the MWM test in T1D mice and T1D mice treated with intermittent leucine deprivation (ILD) (*n* = 8–9). (*n*) The swimming trajectories of T1D and ILD mice during the test period of the MWM test. (o–q) The platform frequence (o) and the percentages of swimming time (*p*) and distance (q) in the original platform area in T1D and ILD mice during the test period of the MWM test (*n* = 8‒9). (r–u) Representative NeuN staining images (r) and the corresponding quantitative data in the CA1 (s), CA3 (t), and DG (u) regions of the hippocampus in T1D and ILD mice (*n* = 7). Scale bar = 100 μm. (v) The mRNA expression levels of synaptic plasticity-related proteins in the hippocampus of T1D and ILD mice by RT-qPCR (*n* = 8). Data were presented as the means ± standard errors. The statistical difference among different groups was analyzed by one-way ANOVA with a post-hoc Dunnett’s test for multiple comparisons, and different lowercase letters indicate statistically significant differences among groups (*p* < 0.05). The statistical difference between two groups was assessed by a two-tailed unpaired Student’s *t*-test, and *p* < 0.05 was considered as a statistically significant difference. Significance level: *p < 0.05; **p < 0.01; ***p < 0.001. The significant of difference in the escape latency during a 4-day training period was evaluated by two-way repeated measures ANOVA: G*T, interaction effect of group and time; G, main effect of group; T, main effect of time.

After 12 weeks of diabetic progression, T1DL mice required significantly more time to locate the hidden platform than CON and T1D mice, as indicated by a prolonged escape latency (Figure S6b, *p *< 0.001). The behavioral parameters calculated from the swimming trajectories (Figure S6c) showed that the platform frequency (Figure S6d) and the proportions of swimming time (Figure S6e) and swimming distance (Figure S6f) within the original platform region were markedly lower in both T1D and T1DL mice relative to CON mice, and this phenomenon was more pronounced in the T1DL mice. These results imply that excessive leucine exacerbated cognitive impairment in T1D mice. Besides, we observed results similar to those at 8 weeks from H&E (Figure S7c), Nissl (Figure S7d), and NeuN (Figures S6g–S6j) staining, indicating that excessive leucine exacerbated hippocampal neuronal injury in T1D mice. At 12 weeks, synaptic plasticity-related markers in the hippocampus were also decreased in T1DL mice relative to T1D mice, particularly PSD95, EGR-1, and c-FOS (Figure S6k).

To further verify the effect of excessive leucine on spatial learning and memory ability in diabetic mice, we conducted a 12-week intermittent leucine deprivation (ILD) experiment, in which T1D mice were fed a leucine-deficient diet every other day (Figure 3l). The results demonstrated that the escape latency during the training period was significantly lower in ILD mice relative to T1D mice (Figure 3m, *p *< 0.001), indicating that a reduction of leucine intake effectively ameliorated learning deficits in T1D mice. Moreover, a reduced leucine intake also significantly alleviated memory deficits in T1D mice, as indicated by the swimming trajectories (Figure 3n), as well as significant increases in the platform frequency (Figure 3o) and the proportions of swimming time (Figure 3p) and swimming distance (Figure 3q) within the original platform region for ILD mice. According to the H&E staining results, we also found that hippocampal neurons were in a disordered arrangement and nuclear shrinkage in different regions of T1D mice, which can be relieved in T1D mice after ILD (Figure S8a). The results from the Nissl and NeuN staining show that ILD mice exhibited notably higher levels of Nissl bodies (Figure S8b) and NeuN-positive areas (Figure 3r–u) in the hippocampal regions than T1D mice, implying that a reduction of leucine intake can alleviate T1D-induced neuronal injury in the hippocampus. Additionally, the hippocampal mRNA levels of synaptic plasticity-related proteins were significantly higher in ILD mice than in T1D mice (Figure 3v). Collectively, these findings confirm that excessive leucine can promote hippocampal neuronal injury and cognitive dysfunction in diabetic mice.

### 2.4. *Akkermansia muciniphila* alleviates neuronal injury and cognitive decline in T1D mice by consuming leucine

To investigate the critical role of the gut microbiota in leucine-mediated DACD, mice were treated with a broad-spectrum antibiotic (ABX) cocktail to deplete the endogenous gut bacteria and administered leucine by oral gavage for 2 weeks, followed by the MWM test (Figure S11a). The plate-counting results show that ABX treatment markedly reduced the levels of both aerobic and anaerobic bacteria in the intestinal contents of the mice (Figures S11b–S11d). Compared with CON mice, the levels of leucine in the intestinal contents were significantly increased in ABX mice and further elevated in ABXL mice (Figure S11e). In the serum and hippocampus, the leucine levels were slightly higher in ABX mice than those in CON mice, but ABXL mice exhibited significantly higher leucine levels relative to other two groups (Figures S11f, S11g). The MWM results revealed that ABXL mice had a significantly longer escape latency than both CON and ABX mice (Figure S11h). As shown by the swimming trajectories, CON and ABX mice spent more of their swimming paths around the target quadrant, while ABXL mice exhibited more peripheral swimming patterns along the pool wall (Figure S11i). The proportions of swimming time (Figure S11j) and swimming distance (Figure S11k) within the original platform region as well as the platform frequency (Figure S11l), were significantly lower in ABXL mice relative to CON and ABX mice. In addition, we observed that neuronal cells were in a disordered arrangement and nuclear shrinkage in the CA1, CA3, and DG regions of the hippocampus in ABXL mice compared with CON and ABX mice (Figure S12a). The results from the Nissl and NeuN staining demonstrated that ABXL mice exhibited notably lower levels of Nissl bodies (Figure S12b) and NeuN-positive areas (Figures S11m-S11p) in the hippocampal regions than CON and ABX mice. Besides, synaptic plasticity-related markers, including SYP (Figure S11q), PSD95 (Figure S11r), EGR-1 (Figure S11s), c-FOS (Figure S11t), and ARC (Figure S11u), in the hippocampus were also significantly decreased in ABXL mice relative to other two groups. Thus it can be seen that depletion of the gut microbiota impaired leucine catabolism, leading to abnormal leucine accumulation in the host and thereby promoting neuronal injury and impairments in learning and memory abilities.

To further explore the associations among the gut bacteria, BCAAs and behavioral parameters, a correlation network analysis was performed in this study (Figure 4a). We found that *Akkermansia* was significantly positively correlated with the platform frequency as well as the proportions of swimming time and swimming distance within the original platform region, but was negatively related to the serum leucine, isoleucine, and valine levels and the hippocampal leucine level (Figure 4a). In addition, T1D mice had notably lower levels of *Akkermansia muciniphila* (AKK, Figure 4b) and branched-chain aminotransferase (BCAT, Figure 4c) in the gut microbiota than CON mice. Based on the KEGG genome database, we identified that AKK bacteria encode BCAT (Figure 4d), suggesting their potential capacity for BCAAs catabolism. *In vitro* experiments showed that the leucine level was markedly reduced after incubation with the AKK bacterial suspension for 10 and 15  min (Figure 4e), confirming that AKK bacteria possess the ability to catabolize leucine. As the liver and muscle are the primary organs of BCAAs metabolism, we measured the expression levels of two key enzymes, involving BCAAs catabolism, namely, BCAT and branched-chain ketoacid dehydrogenase (BCKDH), in the liver and muscle of T1D mice. We found that T1D mice possessed significantly lower levels of BCKDH and BCAT in both liver (Figure S9a and s9b) and muscle (Figure S9c and S9d) than CON mice. Given that leucine is one of the essential amino acids that need to be obtained from the diet, our findings suggest that increased leucine levels in T1D mice may be attributed to reduced leucine catabolic capacity in the gut microbiota and its host.

**Figure 4.**
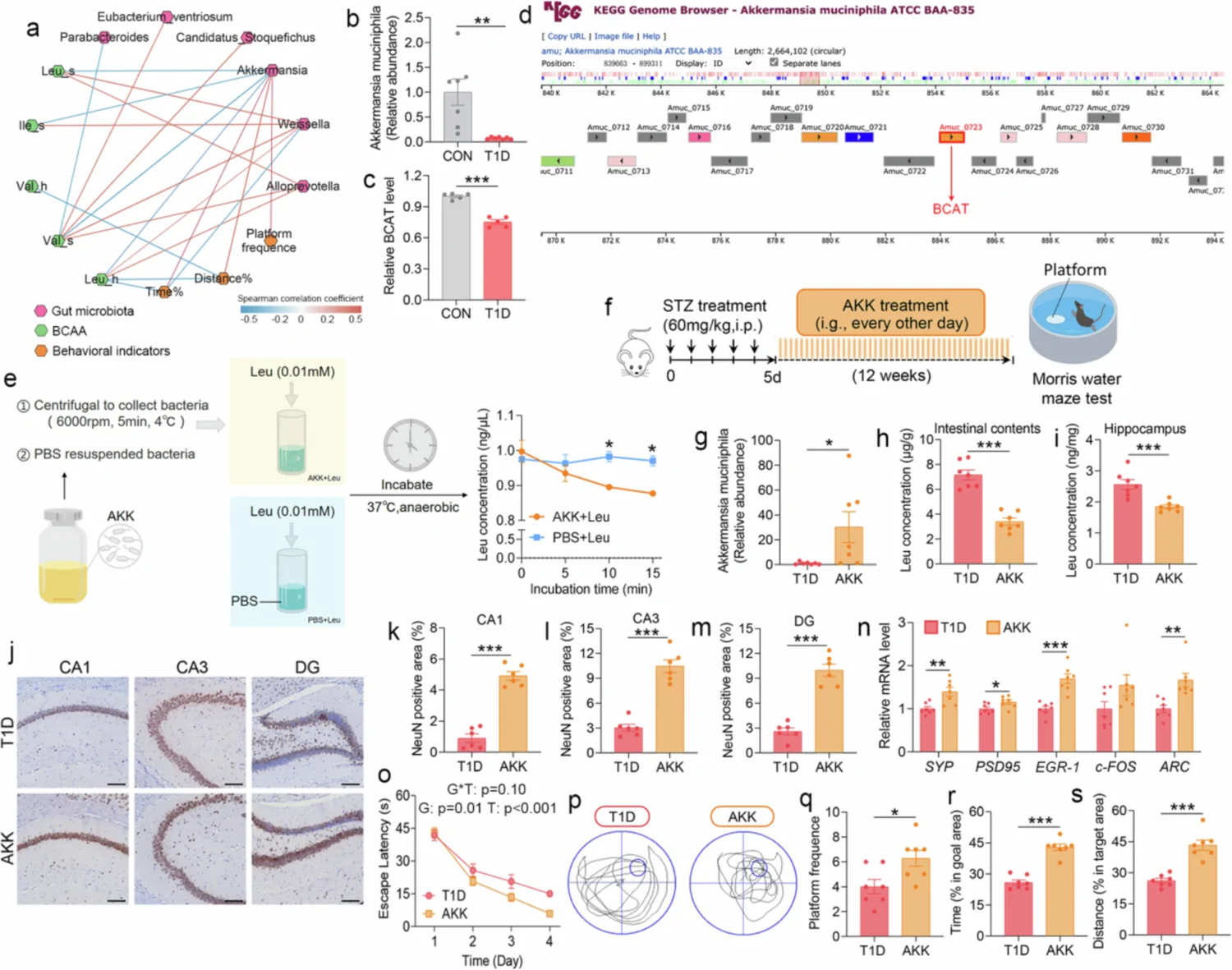
*Akkermansia muciniphila* relieves neuronal injury and cognitive decline in diabetic mice by consuming leucine. (a) Correlation network analysis among the gut microbiota, branched-chain amino acids (BCAAs), and cognitive parameters. (b) Relative abundance of *A. muciniphila* (AKK) in normal control (CON) and type 1 diabetic (T1D) mice by PCR (*n* = 7). (c) Relative mRNA levels of branched-chain aminotransferase (BCAT) in the feces of CON and T1D mice by RT-qPCR (*n* = 5). (d) KEGG genome database showing that AKK bacteria encode BCAT. (e) The co-incubation experiment of leucine with AKK bacteria and the changes in the leucine concentration (*n* = 5). (f) Experimental procedure: T1D mice were orally administered with AKK bacteria every other day for 12 weeks, and then their learning and memory abilities were assessed by using the Morris water maze (MWM) test. (g) Relative abundance of AKK bacteria in T1D mice and T1D mice treated with AKK bacteria by PCR (*n* = 7). (h, i) The concentrations of leucine in the intestinal contents (h) and hippocampus (i) of T1D and AKK mice by LC–MS analysis (*n* = 7). (j–m) Representative NeuN staining images (j) and the corresponding quantitative data in the CA1 (k), CA3 (l), and DG (m) regions of the hippocampus in T1D and AKK mice (*n* = 6). Scale bar = 100 μm. (*n*) The mRNA expression levels of synaptic plasticity-related proteins in the hippocampus of T1D and AKK mice by RT-qPCR (*n* = 7). (o) The escape latency during a 4-day training period of the MWM test in T1D and AKK mice (*n* = 7). (p) The swimming trajectories of T1D and AKK mice during the test period of the MWM test. (q–s) The platform frequency (q) and the percentages of swimming time (r) and distance (s) in the original platform area in T1D and ILD mice during the test period of the MWM test (*n* = 7). Data were presented as the means ± standard errors. The statistical difference between two groups was assessed by a two-tailed unpaired Student’s *t*-test, and *p* < 0.05 was considered as a statistically significant difference. Significance level: *p < 0.05; **p < 0.01; ***p < 0.001. The significant of the difference in the escape latency during a 4-day training period was evaluated by two-way repeated ANOVA: G*T, interaction effect of group and time; G, main effect of group; T, main effect of time.

Subsequently, AKK bacteria were orally administered by gavage to T1D mice every other day for 12 weeks during the course of diabetes progression (Figure 4f). After 12 weeks, AKK bacteria were successfully colonized in the intestinal tract of T1D mice, as indicated by the significantly increased abundance of AKK bacteria in AKK-treated mice (Figure 4g). We also found that treatment with AKK bacteria notably reduced leucine levels in the intestinal contents (Figure 4h) and hippocampus (Figure 4i) of T1D mice. Moreover, exogenous AKK administration markedly attenuated T1D-associated neuronal injury, as indicated by H&E (Figure S10a), Nissl (Figure S10b), and NeuN (Figure 4j‒m) staining. The relative mRNA levels of synaptic plasticity-associated markers were significantly elevated in the hippocampus of T1D mice after AKK treatment (Figure 4n). Furthermore, we evaluated spatial learning and memory ability in mice by the MWM test, and found that treatment with AKK bacteria markedly shortened the escape latency in T1D mice (Figure 4o, *p* = 0.01), indicating an ameliorative learning ability. After AKK treatment, the swimming paths of T1D mice were concentrated within the original platform region (Figure 4p). The platform frequency (Figure 4q) and the proportions of swimming time (Figure 4r) and swimming distance (Figure 4s) within the original platform region were drastically increased in T1D mice fed with AKK bacteria. These results suggest that AKK bacteria lowered host leucine levels through catabolic metabolism, thereby mitigating neuronal injury and cognitive impairment induced by diabetes.

### 2.5. Excessive leucine leads to neuronal injury by overactivating mitophagy

To explore the underlying mechanisms of the promoting effect of excessive leucine on neuronal injury, human neuroblastoma SH-SY5Y cells were administrated with exogenous leucine under HG conditions, followed by transcriptomic analysis and GSEA to identify significantly altered enrichment pathways (Figure 5a). We identified 50 significantly altered enrichment pathways between the high glucose plus leucine (HGL) group and the NG group, 22 between the HG and NG groups, and 23 between the HGL and HG groups (Figure 5b). Relative to the NG group, Venn diagram analysis identified nine commonly altered enrichment pathways in the HGL and HG groups (Figure 5b), and the detailed information is listed in Figure S13. Among these, both HG and HGL treatments were found to induce cell apoptosis (Figure S14). Compared with the NG and HG groups, HGL treatment commonly altered eight enrichment pathways, including mitophagy, as shown in Figures 5b and S13. From the enrichment plots, we found that HGL treatment significantly up-regulated mitophagy in SH-SY5Y cells relative to the NG (Figure 5c) and HG (Figure 5d) groups. Therefore, these findings suggest that neuronal apoptosis occurred under both HG and HGL conditions, whereas mitophagy was specifically induced in the presence of leucine.

**Figure 5.**
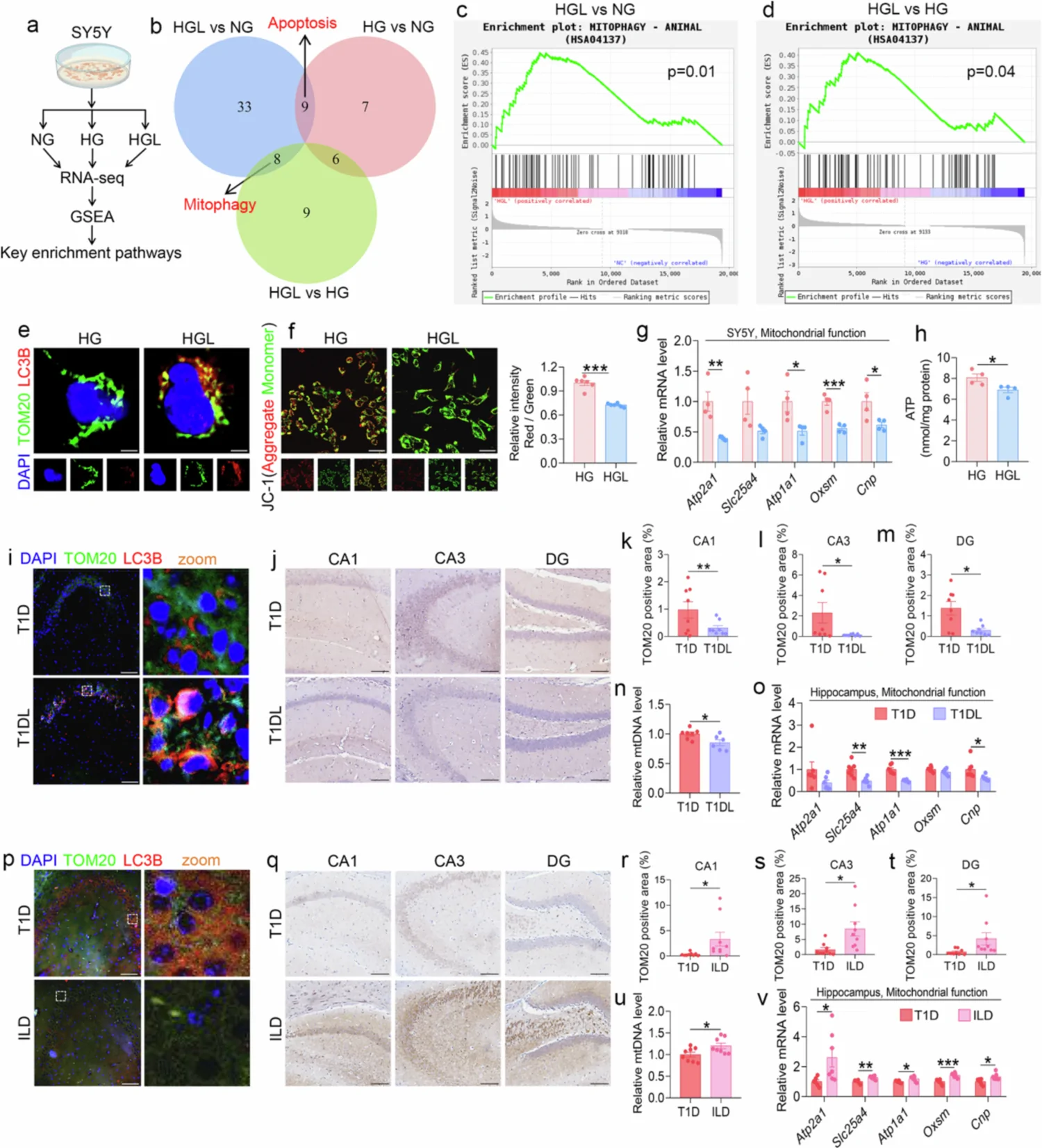
Excessive leucine leads to neuronal injury by overactivating mitophagy. (a) Experimental procedure: Transcriptomic and GSEA analyses were carried out in SH-SY5Y cells to identify notably altered signaling pathways. Group: NG, SH-SY5Y cells cultured under a normal glucose (5.5  mM) condition; HG, SH-SY5Y cells cultured under a high glucose condition; HGL, SH-SY5Y cells treated with leucine under a high glucose condition. (b) Venn diagram illustrating the significantly altered signaling pathways among the NG, HG, and HGL groups. (c, d) The changes in mitophagy between HGL and NG groups (c) and between HGL and HG groups (d). (e) Representative immunofluorescence staining images on the colocalization of TOM20 and LC3B in SH-SY5Y cells after HG and HGL treatments. Scale bar = 5 μm. (f) Representative JC-1 immunofluorescence staining images in SH-SY5Y cells after HG and HGL treatments and the ratio of aggregate to monomer fluorescence (*n* = 6). Scale bar = 50 μm. (g) The mRNA expression levels of mitochondrial function-related proteins in SH-SY5Y cells after HG and HGL treatments by RT-qPCR (*n* = 4). (h) The ATP levels in SH-SY5Y cells after HG and HGL treatments by ATP assay kit (*n* = 4). (i) Representative immunofluorescence staining images on the colocalization of TOM20 and LC3B in the hippocampus of type 1 diabetic (T1D) mice and T1D mice treated with leucine (T1DL). Scale bar = 100 μm. (j–m) Representative TOM20 staining images (j) and the corresponding quantitative data from the CA1 (k), CA3 (l), and DG (m) regions of the hippocampus in T1D and T1DL mice (*n* = 8). Scale bar = 100 μm. (n) Relative mtDNA level in the hippocampus of T1D and T1DL mice by RT-qPCR (*n* = 6‒7). (o) The mRNA expression levels of mitochondrial function-related proteins in the hippocampus of T1D and T1DL mice by RT-qPCR (*n* = 6‒7). (p) Representative immunofluorescence staining images on the colocalization of TOM20 and LC3B in the hippocampus of T1D mice and T1D mice treated with intermittent leucine deprivation (ILD). Scale bar = 100 μm. (q‒t) Representative TOM20 staining images (q) and the corresponding quantitative data from the CA1 (r), CA3 (s), and DG (t) regions of the hippocampus in T1D and ILD mice (*n* = 9). Scale bar = 100 μm. (u) Relative mtDNA levels in the hippocampus of T1D and ILD mice by RT-qPCR (*n* = 8). (v) The mRNA expression levels of mitochondrial function-related proteins in the hippocampus of T1D and ILD mice by RT-qPCR (*n* = 7). Data were presented as the means ±  standard errors. The statistical difference between two groups was assessed by a two-tailed unpaired Student’s *t*-test, and *p* < 0.05 was considered as a statistically significant difference. Significance level: *p < 0.05; **p < 0.01; ***p < 0.001.

To further evaluate mitophagy flux, we assessed the colocalization of the mitochondrial protein TOM20 and the autophagy protein LC3B in SH-SY5Y cells after HG and HGL treatments. The colocalization of TOM20-LC3B was obviously increased in the HGL group than in the HG group (Figure 5e), indicating that excessive leucine triggered the activation of mitophagy. We next evaluated the mitochondrial membrane potential using JC-1 staining, and found that the aggregate to monomer fluorescence ratio was significantly lower in the HGL group than in the HG group (Figure 5f), suggesting that mitochondrial damage occurred in SH-SY5Y cells. The mRNA expression of mitochondrial function-related proteins (Figure 5g) and ATP levels (Figure 5h) were also markedly reduced in the HGL group. In addition, SH-SY5Y cells had a significantly increased level of ROS after HGL treatment (Figure S15). Consistently, in primary neurons, HGL treatment led to a significant decline in the ratio of aggregate to monomer fluorescence (Figure S16a) and a marked increase in ROS levels (Figure S16b). These results indicate that excessive leucine induced the overactivation of mitophagy, thereby leading to mitochondrial damage.

In *in vivo* experiments, exogenous leucine supplementation markedly enhanced TOM20 and LC3B colocalization in the hippocampus of T1D mice (Figure 5i), while the TOM20-positive region was substantially decreased in the CA1, CA3, and DG regions (Figure 5j–m). Additionally, the relative mtDNA level (Figure 5n) and the mRNA expression of mitochondrial function-associated proteins (Figure 5o) were also notably decreased in the hippocampus of T1D mice after leucine treatment. However, after intermittent leucine deprivation (ILD), the colocalization of TOM20 and LC3B in the hippocampus was substantially lower in T1D mice (Figure 5p), and the TOM20-positive region (Figure 5q–t), relative mtDNA level (Figure 5u), as well as the expression of mitochondrial function-related genes (Figure 5v) were significantly elevated. Therefore, these results further verify that excessive leucine resulted in the overactivation of mitophagy and mitochondrial dysfunction in the hippocampus of T1D mice.

### 2.6. Leucine facilitates neuronal mitophagy by interaction with TBK1

In this study, we combined drug affinity responsive target stability (DARTS) and Astral DIA-based quantitative proteomics to identify potential molecular targets mediating leucine-induced overactivation of neuronal mitophagy (Figure 6a). As shown in Figure 6b, the proteins were separated by SDS-PAGE and stained with Coomassie brilliant blue. The gel region from 72 to 100 kDa was excised for LC‒MS proteomics analysis, resulting in the identification of 2,586 candidate target proteins (Figure 6c). We then cross-referenced these candidates with mitophagy- and cognitive dysfunction-related proteins from the GeneCards database via Venn diagram analysis to identify common proteins (Figure 6c). Ultimately, three potential protein targets were selected, including TBK1, MFN2, and DNM1L (Figure 6d), of which TBK1 had the highest abundance and identification accuracy (Figure S17).

**Figure 6.**
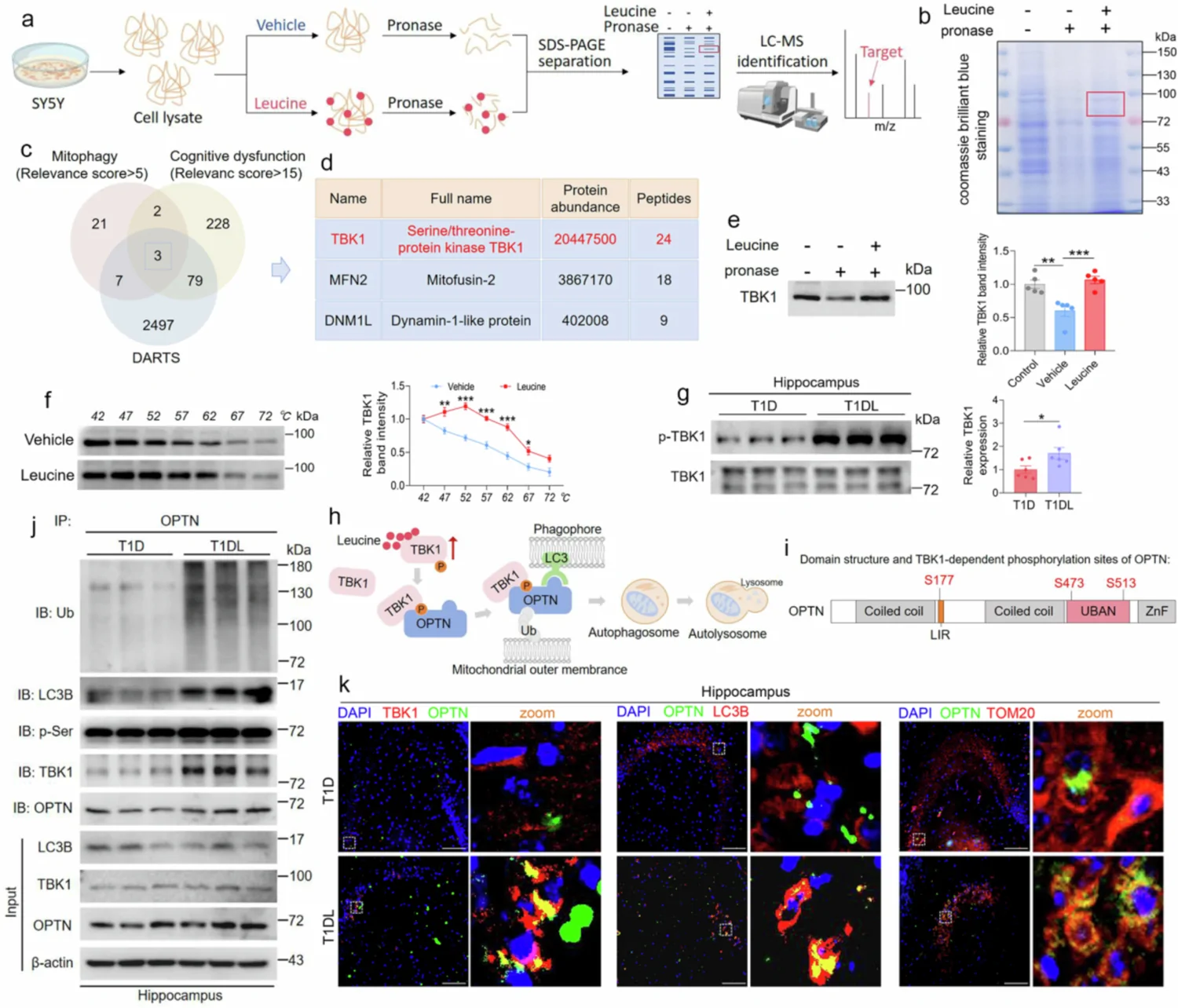
Leucine facilitates neuronal excessive mitophagy by interaction with TBK1. (a) Experimental procedure: The cell lysate of SH-SY5Y was treated with leucine and digested by pronase, and then proteins were separated by SDS–PAGE and stained with Coomassie Brilliant Blue. Subsequently, the gel region of interest was excised for Astral DIA-based quantitative proteomics to identify potential molecular targets of leucine. (b) SDS‒PAGE separation and Coomassie brilliant blue staining. (c, d) Proteins identified from proteomics were cross-referenced with mitophagy- and cognitive dysfunction-related proteins from the GeneCards database via Venn diagram analysis. (e) Drug affinity responsive target stability (DARTS) and the corresponding quantitative results (*n* = 5). (f) Cell thermal shift assay (CETSA) and the corresponding quantitative results (*n* = 5). (g) The phosphorylation level of TBK1 was analyzed by Western blotting in the hippocampus of type 1 diabetic (T1D) mice and T1D mice treated with leucine (T1DL) (*n* = 6). (h) Schematic diagram of leucine-mediated excessive mitophagy: Leucine promotes the phosphorylation of TBK1 and, in turn, phosphorylates OPTN, thereby enhancing the binding capacity of OPTN to LC3 and Ub chains and then initiating mitophagy. (i) Domain structure and TBK1-dependent phosphorylation sites of OPTN, including S177, S473, and S513. (j) Co-immunoprecipitation (Co-IP) experiments showing an increased level of the serine phosphorylation (*p*-Ser) of OPTN and an enhanced interaction of OPTN with TBK1, ubiquitin (Ub), and LC3B in the hippocampus of T1DL mice. (k) Representative immunofluorescence staining images on the colocalization of OPTN with TBK1, LC3B, and TOM20 in the hippocampus of T1DL mice. Scale bar = 100 μm. Data were presented as the means ±  standard errors. The statistical difference among different groups was analyzed by one-way ANOVA with a post-hoc Dunnett’s test for multiple comparisons, and different lowercase letters indicate statistically significant differences among groups (*p* < 0.05). The statistical difference between two groups was assessed by a two-tailed unpaired Student’s *t*-test, and *p* < 0.05 was considered as a statistically significant difference. Significance level: *p < 0.05; **p < 0.01; ***p < 0.001.

Furthermore, DARTS and cell thermal shift assay (CETSA) experiments were employed to further confirm the interaction between leucine and TBK1. The DARTS results revealed that leucine notably reduced the degradation of TBK1 by pronase (Figure 6e), and the CETSA assays also showed that leucine prevented the degradation of TBK1 with increasing temperature from 42 to 72 °C (Figure 6f). Given that TBK1 is a protein kinase, we next investigated the effect of leucine on its phosphorylation. Exogenous leucine supplementation markedly increased the phosphorylation level of hippocampal TBK1 in T1D mice (Figure 6g). TBK1 has been reported to phosphorylate OPTN at S177, S473, and S513, thereby enhancing the binding capacity of OPTN to LC3 and Ub chains and initiating mitophagy,^21^ as shown in Figure 6h and i. To further validate the involvement of leucine in TBK1-driven mitophagy, co-immunoprecipitation (Co-IP) experiments were performed in this study. The results demonstrate that exogenous leucine supplementation markedly increased the serine phosphorylation (*p*-Ser) of OPTN in the hippocampus of T1D mice and enhanced its interactions with TBK1, ubiquitin (Ub), and LC3B (Figure 6j). The immunostaining results also revealed that leucine treatment markedly increased the colocalization of OPTN with TBK1, LC3B, and TOM20 in the hippocampus of T1D mice (Figure 6k). Besides, similar results were observed in SH-SY5Y cells, as illustrated in Figure S18. Hence, our findings elucidate that excessive leucine activates aberrant mitophagy in neurons by interaction with TBK1.

### 2.7. The overactivation of neuronal mitophagy by leucine is TBK1-dependent

To verify the essential role of TBK1 in leucine-induced excessive mitophagy in neurons, we used a brain-targeted AAV delivery system (shTBK1) to knock down TBK1 in the hippocampus of T1D mice (Figure 7a). T1D mice treated with shTBK1 exhibited significantly lower levels of TBK1 at both the mRNA (Figure 7b) and protein (Figure 7c) levels in the hippocampus compared with that treated with the empty vector (shNC), indicating that TBK1 was successfully knocked down. The results from immunofluorescence staining demonstrated that leucine treatment markedly enhanced the colocalization of LC3B and TOM20 in the hippocampus of T1D mice treated with shNC, whereas TBK1 knockdown abolished this effect, as indicated by no significant changes in LC3B-TOM20 colocalization in shTBK1-treated T1D mice after leucine administration (Figure 7d). Leucine treatment drastically reduced TOM20-positive region in all hippocampal regions of shNC-treated mice, but no significant alterations were obtained in TBK1-deficient T1D mice (Figure S19). In addition, we found that leucine treatment significantly reduced mtDNA levels (Figure 7e) and the mRNA expression of mitochondrial function-related proteins (Figure 7f) in the hippocampus of shNC-treated T1D mice, whereas no significant alterations were observed in TBK1-deficient T1D mice. Consistently, H&E (Figure S20a), Nissl (Figure S20b), and NeuN (Figure 7g–j) staining also showed that exogenous leucine treatment notably exacerbated hippocampal neuronal injury in shNC-treated T1D mice, but not in TBK1-deficient T1D mice. We found a similar result for the relative mRNA expression of synaptic plasticity-related proteins, as illustrated in Figure 7k. The MWM results demonstrated that leucine treatment significantly increased escape latency during the training phase in shNC-treated T1D mice, whereas this phenomenon was abolished in TBK1-deficient T1D mice (Figure 7l, *p* < 0.001). During the testing phase, the swimming paths of the TBK1-deficient T1D mice were still concentrated in the original platform area relative to those of the other groups (Figure S21). The platform frequency (Figure 7m) and the proportions of swimming time (Figure 7n) and swimming distance (Figure 7o) within the original platform region were significantly lower in shNC-treated T1D mice under leucine administration, but not in TBK1-deficient T1D mice. Therefore, we suggest that TBK1 mediated the detrimental effects of excessive leucine on learning and memory abilities through the overactivation of neuronal mitophagy in T1D mice.

**Figure 7.**
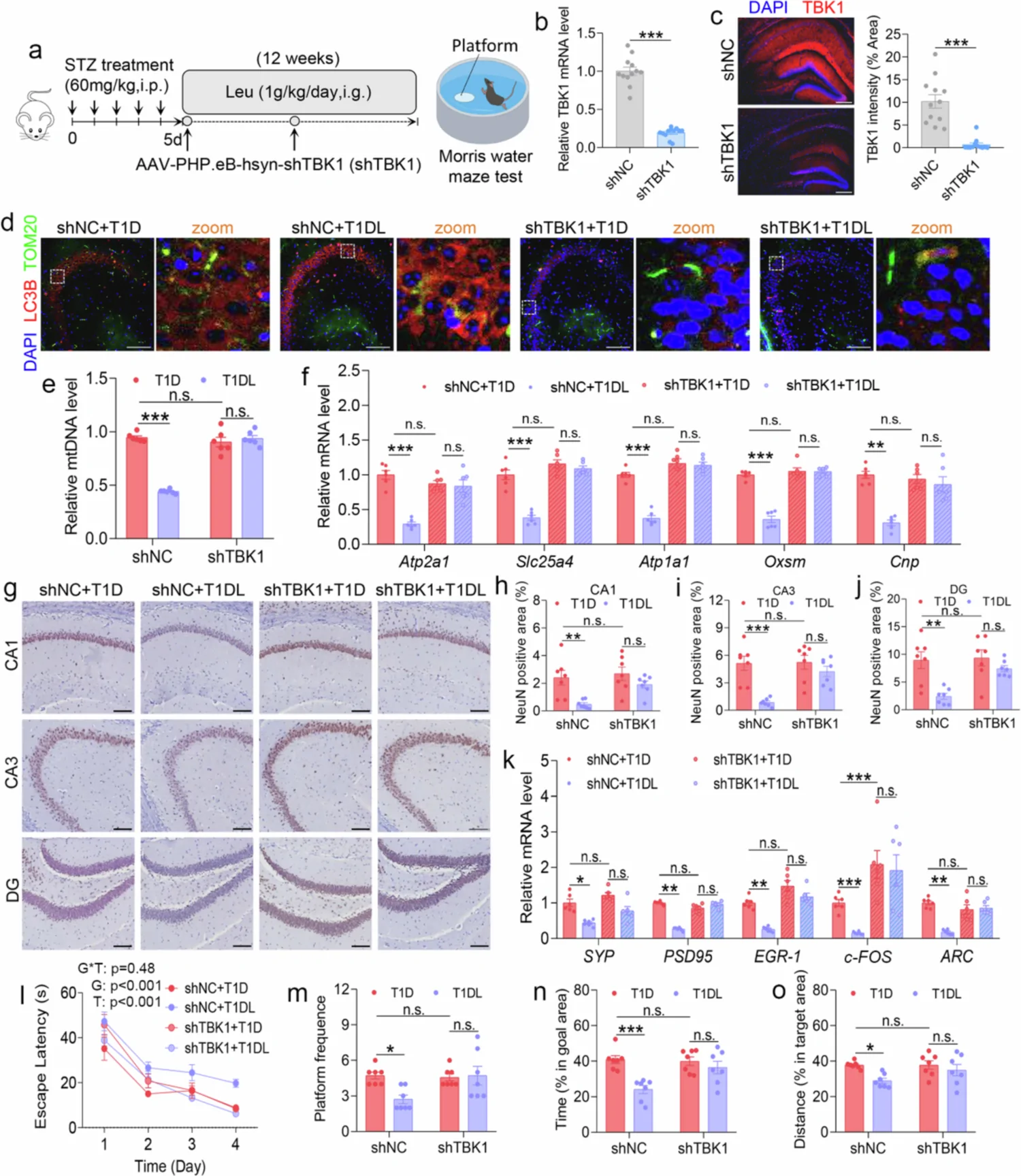
The overactivation of neuronal mitophagy by leucine is TBK1-dependent. (a) Experimental procedure: Herein, type 1 diabetic (T1D) mice were established by intraperitoneal injection of streptozotocin (STZ) and treated with leucine for 12 weeks. During this period, T1D mice were administered a brain-targeted AAV delivery system (shTBK1) to knock down TBK1 expression in the hippocampus. Subsequently, the learning and memory abilities of mice were evaluated by using the Morris water maze (MWM) test. (b) Relative mRNA expression level of TBK1 in the hippocampus of T1D mice treated with empty vector (shNC) and shTBK1 by RT-qPCR (*n* = 12). (c) Representative immunofluorescence staining images and the corresponding quantitative results of TBK1 expression in the hippocampus of T1D mice treated with shNC and shTBK1 (*n* = 12). Scale bar = 50 μm. (d) Representative immunofluorescence staining images on the colocalization of TOM20 and LC3B in the hippocampus of T1D mice and leucine-fed T1D (T1DL) mice treated with shNC and shTBK1. Scale bar = 100 μm. (e) Relative mtDNA level in the hippocampus of T1D and T1DL mice treated with shNC and shTBK1 by RT-qPCR (*n* = 6). (f) The mRNA expression levels of mitochondrial function-related proteins in the hippocampus of T1D and T1DL mice treated with shNC and shTBK1 by RT-qPCR (*n* = 6). (g–j) Representative NeuN staining images (g) and the corresponding quantitative data in the CA1 (h), CA3 (i), and DG (j) regions of the hippocampus in T1D and T1DL mice treated with shNC and shTBK1 (*n* = 7-8). Scale bar = 100 μm. (k) The mRNA expression levels of synaptic plasticity-related proteins in the hippocampus of T1D and T1DL mice treated with shNC and shTBK1 by RT-qPCR (*n* = 6). (l) The escape latency during a 4-day training period of the MWM test in T1D and T1DL mice treated with shNC and shTBK1 (*n* = 7–8). (m–o) The platform frequency (m) and the percentages of swimming time (*n*) and distance (o) in the original platform area in T1D and T1DL mice treated with shNC and shTBK1 during the test period of the MWM test (*n* = 7–8). Data were presented as the means ±  standard errors. The statistical difference between two groups was assessed by a two-tailed unpaired Student’s *t*-test, and *p* < 0.05 was considered as a statistically significant difference. The statistical difference, with multiple groups, was performed by two-way ANOVA followed by the Sidak post hoc test. Significance level: n.s., no significant difference; *p < 0.05; **p < 0.01; ***p < 0.001. The significant of difference in the escape latency during a 4-day training period was evaluated by two-way repeated measure ANOVA: G*T, interaction effect of group and time; G, main effect of group; T, main effect of time.

In *in vitro* experiments, we silenced TBK1 in SH-SY5Y cells via siRNA transfection, and all three siRNA sequences significantly reduced TBK1 expression at both the mRNA (Figure S22a) and protein (Figures S22b and S22c) levels. Among them, the siRNA sequence 2# with relatively high transfection efficiency was selected for subsequent experiments. In SH-SY5Y cells transfected with the empty vector (si-NC), treatment with high glucose and leucine (HGL) drastically increased the colocalization of LC3B and TOM20 (Figure S22d). However, TBK1 knockdown abolished this effect, as TBK1-deficient SH-SY5Y cells showed no LC3B-TOM20 colocalization under an HGL condition (Figure S22d). The JC-1 staining showed a significant reduction in the aggregate-to-monomer fluorescence ratio in si-NC-treated SH-SY5Y cells, whereas TBK1 knockdown effectively counteracted this decrease (Figure S22e). After TBK1 knockdown, treatment with HGL did not reduce the mRNA expression levels of mitochondrial function-associated proteins in SH-SY5Y cells (Figure S22f). Additionally, si-NC-treated SH-SY5Y cells had a significantly increased levels of ROS after HGL treatment, but this phenomenon did not occurred in TBK1-deficient SH-SY5Y cells (Figure S22g). The results from *in vitro* assays further confirm that the overactivation of mitophagy by leucine was mediated in a TBK1-dependent manner.

### 2.8. High leucine levels are correlated with hippocampal atrophy and cognitive deficits in diabetic patients

To inspect whether T2D possesses similar pathological characteristics, we established a T2D mouse model by combining a high-fat diet with STZ induction and assessed learning and memory abilities after 20 weeks of diabetic progression (Figure 8a). Compared with CON mice, T2D mice exhibited significantly elevated fasting blood glucose (Figure 8b) and insulin (Figure 8c) levels as well as increased HOMA-IR values (Figure 8d), which are consistent with the clinical phenotype of patients with T2D. During the training period, the escape latency was significantly longer in T2D mice relative to CON mice (Figure 8e, *p* = 0.02), implying an impaired learning ability. Moreover, T2D mice exhibited lower memory ability than CON mice, as indicated by the swimming trajectories (Figure 8f), as well as significantly decreased levels of platform frequency (Figure 8g) and the proportions of swimming time (Figure 8h) and swimming distance (Figure 8i) within the original platform region. Similar to T1D mice, we also found that T2D mice exhibited a marked reduction in AKK bacteria in the gut (Figure 8j), accompanied by significantly elevated leucine levels in both serum (Figure 8k) and hippocampus (Figure 8l). Relative to CON mice, however, the mRNA expression levels of BCKDH (Figures S23a and S23b) and BCAT (Figures S23c and S23d) showed no significant changes in both liver and muscle of T2D mice. According to the hippocampal H&E staining, we found that T2D mice displayed the same phenomenon as T1D mice, characterized by disorganized neuronal arrangement and nuclear shrinkage in the CA1, CA3, and DG regions (Figure S24a). Relative to CON mice, we found that T2D mice had notably lower levels of Nissl bodies (Figure S24b) and NeuN-positive areas (Figure 8m–p) in different hippocampal regions, indicating that neuronal injury occurred in T2D mice. In addition, the relative mRNA levels of synaptic plasticity-associated markers in the hippocampus were also significantly lower in T2D mice than in CON mice (Figure 8q). Besides, an obvious increase in the colocalization of TOM20 and LC3B was detected in the hippocampus of T2D mice compared with CON mice (Figure 8r). However, T2D mice had significantly lower levels of TOM20-positive areas in the CA1, CA3, and DG regions of the hippocampus than CON mice (Figure 8s–v). The relative mtDNA level (Figure 8w) and the mRNA expression of mitochondrial function-related markers (Figure 8x) were notably decreased in the hippocampus of T2D mice. Collectively, these findings suggest that T2D-associated cognitive decline might also be attributed to neuronal injury, lower AKK bacterial abundance, higher leucine levels, and overactivation of mitochondrial autophagy.

**Figure 8.**
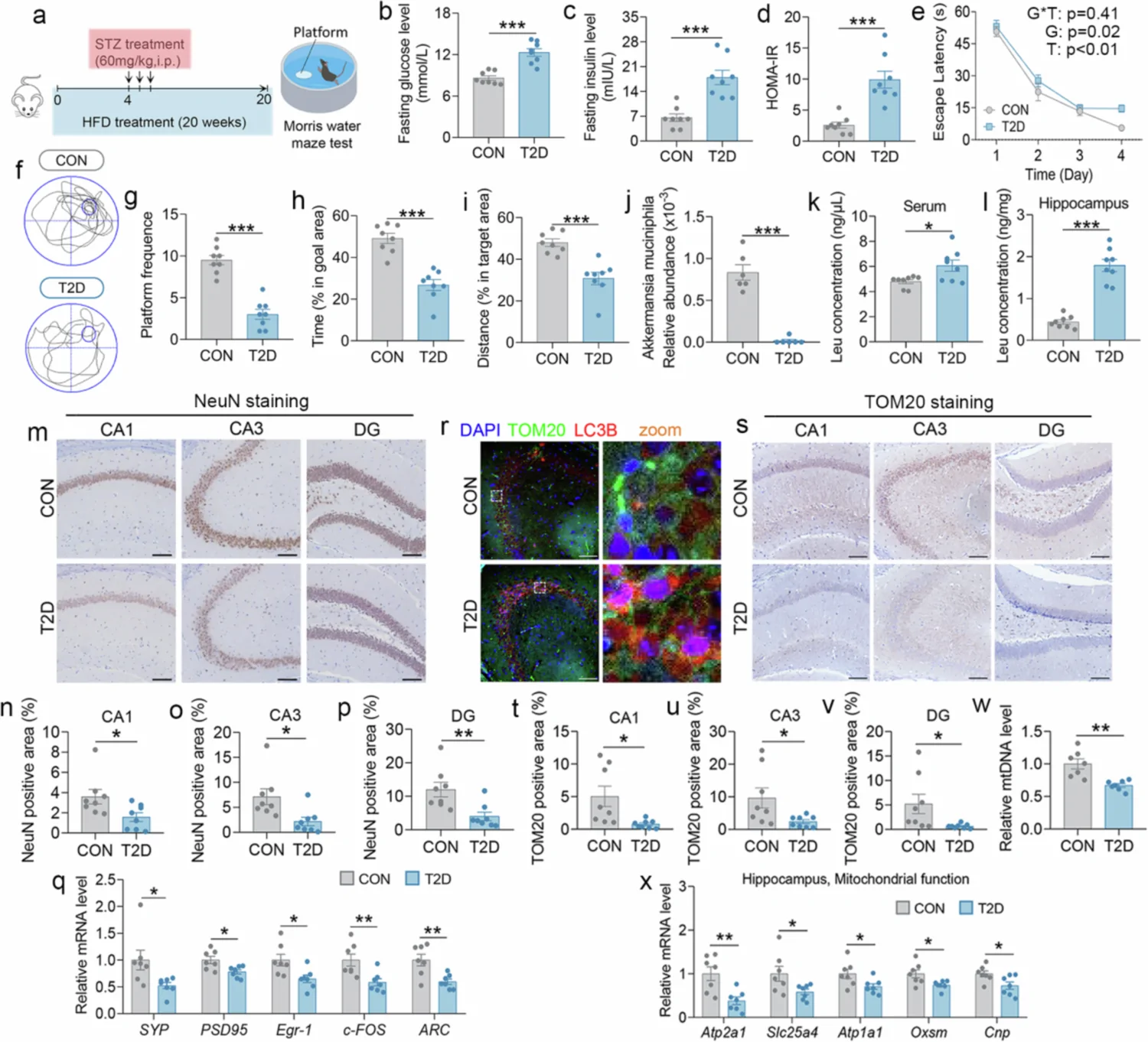
Type 2 diabetic mice exhibit neuronal injury and cognitive decline in the later stage, accompanied by higher leucine and excessive mitophagy. (a) Experimental procedure: In this study, type 2 diabetic (T2D) mice were established by combining a high-fat diet with streptozotocin (STZ) induction, and their learning and memory abilities were evaluated by using the Morris water maze (MWM) test after 20 weeks of diabetic progression. (b-d) The levels of fasting glucose (b), fasting insulin (c), and HOMA-IR (d) in the serum of normal control (CON) and T2D mice (*n* = 8). (e) The escape latency during a 4-day training period of the MWM test in CON and T2D mice (*n* = 8). (f) The swimming trajectories of CON and T2D mice during the test period of the MWM test. (g–i) The platform frequency (g) and the percentages of swimming time (h) and distance (i) in the original platform area in CON and T2D mice during the test period of the MWM test (*n* = 8). (j) Relative abundance of *A. muciniphila* in the feces of CON and T2D mice by PCR (*n* = 6). (k, l) The concentrations of leucine in the serum (k) and hippocampus (l) of CON and T2D mice by LC–MS analysis (*n* = 8). (m–p) Representative NeuN staining images (m) and the corresponding quantitative data in the CA1 (n), CA3 (o), and DG (p) regions of the hippocampus in CON and T2D mice (*n* = 8). Scale bar = 100 μm. (q) The mRNA expression levels of synaptic plasticity-related proteins in the hippocampus of CON and T2D mice by RT-qPCR (*n* = 7). (r) Representative immunofluorescence staining images on the colocalization of TOM20 and LC3B in the hippocampus of CON and T2D mice. Scale bar = 100 μm. (s–v) Representative TOM20 staining images (s) and the corresponding quantitative data in the CA1 (t), CA3 (u), and DG (v) regions of the hippocampus in CON and T2D mice (*n* = 8). Scale bar = 100 μm. (w) Relative mtDNA level in the hippocampus of CON and T2D mice by RT-qPCR (*n* = 7). (x) The mRNA expression levels of mitochondrial function-related proteins in the hippocampus of CON and T2D mice by RT-qPCR (*n* = 7). Data were presented as the means ±  standard errors. The statistical difference between two groups was assessed by a two-tailed unpaired Student’s *t*-test, and *p* < 0.05 was considered as a statistically significant difference. Significance level: *p < 0.05; **p < 0.01; ***p < 0.001. The significant of difference in the escape latency during a 4-day training period was evaluated by two-way repeated measure ANOVA: G*T, interaction effect of group and time; G, main effect of group; T, main effect of time.

To further explore the association between AKK bacteria-mediated leucine catabolism and cognition from a clinical perspective, we recruited a cohort of volunteers, including 25 healthy controls, 19 patients with T1D, and 25 patients with T2D, assessed their cognitive functions, and then collected their serum and feces samples for analysis (Figure 9a). Their clinical characteristics were listed in Table S1, where we can see that all diabetic patients had a disease duration of more than 8 y on average, with mean fasting blood glucose levels exceeding 8 mmol/L. Besides, T1D patients possessed a lower level of BMI than healthy subjects, and T2D patients (Table S1). However, no significant differences were obtained among different groups in age, sex, and the proportions of smoking, alcohol consumption and hypertension (Table S1). Relative to healthy subjects, both T1D and T2D patients had a significantly reduced abundance of AKK bacteria in the feces (Figure 9b) and increased level of leucine in the serum (Figure 9c). We also found that higher abundances of AKK bacteria were associated with lower leucine levels (Figure 9d), suggesting a potential role of AKK bacteria in leucine catabolism. Of note, cognitive function scores, including MoCA and MMSE, were positively correlated with AKK bacteria (Figure 9e and f) but negatively related to serum leucine levels (Figure 9g and h). In addition, we recruited a cohort of 145 patients with T2D, assessed their cognitive functions, and then collected serum samples for leucine determination (Figure 9i). These patients had an average diabetes duration of more than 6 y, with mean fasting blood glucose levels exceeding 8 mmol/L (Table S1). The results further validated that serum leucine levels were significantly negatively related to MoCA (Figure 9j, R = −0.3449, *P* < 0.001) and MMSE (Figure 9j, R = −0.3387, *P *< 0.001) scores in T2D patients. In this cohort, 13 patients underwent magnetic resonance imaging (MRI) examination, and then their hippocampal volumes were quantified and correlated with serum leucine levels. We found that higher serum leucine concentrations were associated with reduced hippocampal volumes in patients with T2D (Figure 9l, R = −0.5659, *P* = 0.0473). Thus, these clinical data imply that reduced AKK bacteria abundance and elevated leucine levels are risk factors for cognitive decline in diabetic patients.

**Figure 9.**
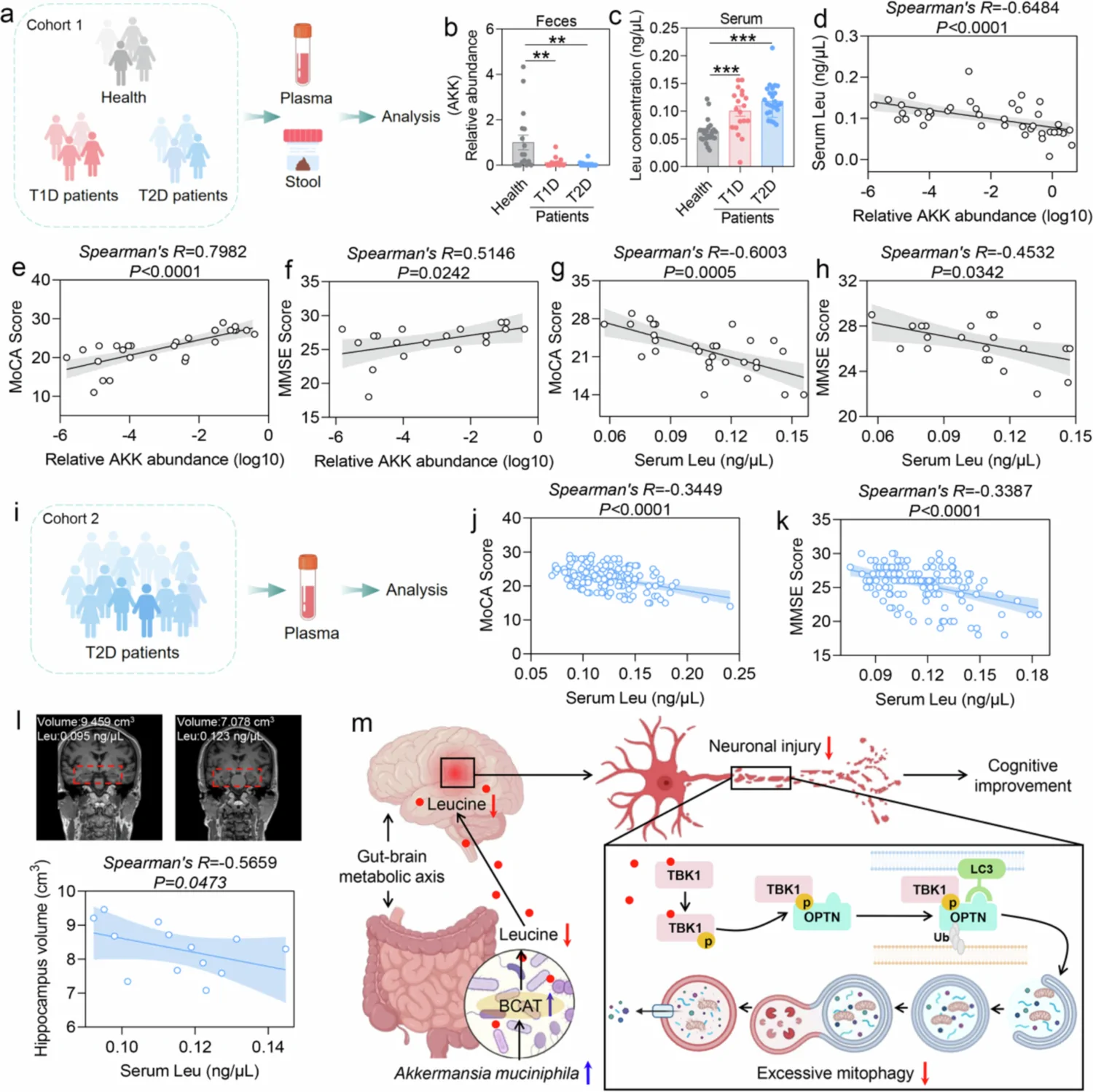
High leucine levels are associated with hippocampal atrophy and cognitive deficits in diabetic patients. (a) Experimental procedure: In cohort 1, we recruited a cohort of volunteers, including healthy controls (*n* = 25), patients with T1D (*n* = 19), and patients with T2D (*n* = 25), assessed their cognitive functions, and then collected their serum and feces samples for analysis. (b) Relative abundance of *Akkermansia muciniphila* (AKK) in the feces of healthy individuals, T1D patients, and T2D patients by PCR. (c) Leucine concentrations in the serum of healthy individuals, T1D patients, and T2D patients by LC-MS analysis. (d) Spearman correlation analysis between serum leucine and AKK bacteria. (e, f) Spearman correlations of AKK bacteria with MoCA (e) and MMSE (f) scores. (g, h) Spearman correlations of serum leucine with MoCA (e) and MMSE (f) scores. (i) Experimental procedure: In cohort 1, we recruited a cohort of patients with T2D (*n* = 145), assessed their cognitive functions, and then collected serum samples for analysis. (j, k) Spearman correlations of serum leucine with MoCA (j) and MMSE (k) scores. (l) Representative magnetic resonance imaging (MRI) of T2D patients and Spearman correlation analysis between serum leucine and hippocampus volume. The hippocampal region is highlighted with a red box. (m) Schematic diagram of the novel gut‒brain metabolic axis mechanism by which AKK bacteria alleviate cognitive decline in diabetes. Data were presented as the means ±  standard errors. The statistical difference between two groups was assessed by a two-tailed unpaired Student’s *t*-test, and *p* < 0.05 was considered as a statistically significant difference. Significance level: **p < 0.01; ***p < 0.001.

## 3. Discussion

Herein, we elucidated a novel gut‒brain metabolic axis mechanism by which AKK bacteria improve cognitive decline in diabetes patients (Figure 9m). First, we found that both diabetic mice and patients displayed a marked reduction in the abundance of AKK bacteria, accompanied by elevated leucine levels. Both leucine supplementation and deprivation studies showed that excessive leucine accelerated and exacerbated cognitive decline in diabetic mice. Moreover, our results verified that AKK bacteria possess the capacity to catabolize leucine and exogenous supplementation with AKK bacteria reduced systemic leucine levels and mitigated cognitive decline in diabetic mice. Subsequently, we revealed that excessive leucine interacted with TBK1, thereby triggering neuronal mitophagy overactivation and resulting in neuronal injury. Finally, we reported that elevated leucine levels were significantly associated with hippocampal atrophy and cognitive impairment in patients with diabetes.

Previous studies have reported that the leucine level is considerably increased in both diabetic animal models^22‐24^ and patients.^25^ ^,^ ^26^ In the early stage of diabetes, BCAAs, especially leucine, can induce insulin resistance through multiple mechanisms, including the persistent activation of mTORC1,^27^ mitochondrial dysfunction,^28^ and the overactivation of hypothalamic AgRP neurons.^29^ During the progression of diabetes, elevated leucine levels promoted the onset and development of diabetes-related complications. For example, excess BCAAs increased cardiac LAT1 expression via inhibiting FGF21 production, thereby leading to increased levels of BCAAs in myocardial cells and eventually causing mitochondrial damage and diabetic cardiomyopathy.^30^ Asakura et al. also reported that the accumulation of leucine induced cardiac hypertrophy via the activation of mTORC1 in diabetic mice.^31^ BCAAs decreased lipogenesis and autophagy in the hepatocyte to exacerbate hepatic lipotoxicity in diabetic mice.^32^ Besides, BCAAs catabolic defects have been verified as a risk factor for diabetic kidney disease by regulating metabolic reprogramming^33^ ^,^ ^34^ and enhancing inflammation.^35^ Notably, however, the association between high leucine and DACD and its underlying mechanisms remains unexplored. Herein, we revealed that elevated leucine levels are closely associated with DACD in both mouse models and humans. Through exogenous leucine intake and deprivation experiments, we further confirmed that high leucine served as a critical driving factor in the of DACD in mice. Elevated leucine levels have also been observed in Alzheimer's disease (AD) mouse models^21^ and patients,^36^ so we speculate that sustained elevation of leucine might be a key factor linking the shared pathogenic mechanism underlying AD and DACD. In this study, we furthermore revealed that excessive leucine triggered the overactivation of neuronal mitophagy and caused neuronal injury. For the first time, we identified TBK1 as a direct molecular target of leucine via target screening and knockdown experiments. Previous studies have identified TBK1 as an important regulator of mitophagy.^37‐39^ Herein, we elucidated that leucine increased the phosphorylation of TBK1 and, in turn, phosphorylated the autophagy adaptor OPTN, thereby enhancing the binding capacity of OPTN to LC3 and Ub chains and initiating excessive mitophagy in neurons.

Previous studies have revealed that the elevated levels of BCAAs in diabetes were attributed to disorders in glycolysis, fatty acid oxidation, and BCAAs transamination.^40^ Given that BCAAs are essential amino acids derived from the daily diet, our findings indicate that the reduced capacity of the gut microbiota to catabolize leucine could also be one of the contributing factors to high leucine in diabetes. Beyond diabetes, dysfunctional BCAAs catabolism has been reported to involve in the onset and development of multiple diseases including heart failure with preserved ejection fraction,^41^ sarcopenia,^42^ aging,^43^ mechanical pain,^44^ tumor proliferation,^45^ kidney injury,^46^ cancer,^47^ and cardiac ischemia-reperfusion injury.^48^ Therefore, enhancing leucine catabolism can effectively ameliorate metabolic dysfunction and insulin resistance in diabetes,^49^ ^,^ ^50^ while exerting similar beneficial effects in other diseases, such as acute kidney injury,^51^ cardiovascular-kidney-metabolic syndrome,^52^ and heart failure.^53^ We propose that gut microbial leucine catabolism may also represent a novel target for the prevention and management of DACD.

Notably, herein we found that the decreased abundance of AKK bacteria in both diabetic mice and patients was significantly associated with elevated leucine levels. Since AKK bacteria possess the capacity to catabolize leucine, we sought to enhance leucine catabolism in diabetic mice by exogenous supplementation with AKK bacteria. As expected, treatment with AKK bacteria markedly reduced leucine levels, thereby alleviating hippocampal neuronal injury and cognitive decline in diabetic mice. An interesting study conducted by Qiao et al. reported that the administration of the gut *Parabacteroides merdae* alleviated atherosclerotic lesions in obese mice by enhancing intestinal BCAAs degradation.^54^ Currently, limited studies have explored the strategy of augmenting intestinal BCAAs catabolism via specific gut microbes for the prevention and management of diseases. Yet, such a microbiota-targeted metabolic intervention may provide a novel paradigm for disease treatment in the future. AKK bacteria have been reported to improve cognitive deficits resulting from diverse etiological factors by regulating the serotonin pathway in mice with liver injury,^55^ inhibiting neuroinflammation in aged mice,^56^ and modulating microglial engulfment of synapses in sleep-deprived mice.^57^ Gong et al. demonstrated that AKK bacteria improved exploratory behavior in diabetic mice by reducing the detrimental microbial metabolite *δ*-valerobetaine, promoting carnitine biosynthesis, limiting lipid droplet accumulation in microglia, and then suppressing neuroinflammation.^58^ Herein, a new gut‒brain metabolic axis mechanism mediated by AKK bacteria to relieve the development of DACD was reported through enhancing intestinal leucine catabolism, reducing host leucine levels, and suppressing neuronal excessive mitophagy. Therefore, we hold the opinion that AKK bacteria can serve as psychobiotics that exert neuroprotective effects by regulating the gut‒brain metabolic axis, thereby providing a gut-targeted strategy for the management of brain disorders.

In conclusion, we reported that a reduced abundance of AKK bacteria and elevated leucine levels are risk factors that contribute to the development of DACD in both diabetic mice and patients. Excessive leucine interacted with TBK1 to promote the overactivation of mitophagy in neurons, thereby leading to neuronal injury and DACD. Moreover, supplementation with AKK bacteria enhanced intestinal leucine catabolism, lowered systemic leucine levels, and mitigated high-leucine-induced neuronal injury and DACD. This study not only uncovers a new gut‒brain metabolic axis mechanism that drives DACD, but also provides a potential strategy for its prevention and treatment.

## 4. Materials and methods

### 4.1. Animals

Six-week-old male C57BL/6 mice with body weights ranging from 18 to 22 g were supplied by Vital River Laboratory Animal Technology Co., Ltd. (Beijing, China). All animals were housed in specific pathogen-free (SPF) cages at the Laboratory Animal Center of Wenzhou Medical University (WMU, Wenzhou, China). The animal room was maintained under standardized environmental conditions, with a temperature of 22 ± 2 °C, relative humidity of 55 ± 5%, and a 12-h light/12-h dark cycle, with lights turned on at 8:00 a.m. Sterile standard chow and water were provided ad libitum throughout the study. Before the start of the experiments, the mice were acclimated to the housing conditions for one week. All animal procedures complied with the Guide for the Care and Use of Laboratory Animals and were approved by the Institutional Animal Care and Use Committee of WMU (Approval No. xmsq2024-0754).

### 4.2. Participant recruitment and sample collection

In this study, healthy volunteers and diabetic patients were recruited after matching for age and sex, and excluding individuals with neurological or psychiatric disorders. Diabetic patients had a disease duration of at least 1  y, and their baseline characteristics are presented in Table S1. Serum and fecal samples were obtained from The First Affiliated Hospital of Wenzhou Medical University (WMU). This study was conducted according to the principles of the Declaration of Helsinki and approved by the Ethics Committee of The First Affiliated Hospital of WMU (ID: KY2021-153), and written informed consents were obtained from all participants.

### 4.3. Establishment of type 1 diabetic (T1D) mouse model

After a one-week acclimation period, the mice were weighed and randomly assigned to either the normal control (CON, *n* = 7) group or the diabetic group (*n* = 15). After 12 h of fasting, the mice in the diabetic group received intraperitoneal injections of streptozotocin (STZ, S8050, Solarbio, Beijing, China) dissolved in citrate buffer (0.1 M, pH = 4.5) at 60  mg/kg once daily for 5 consecutive days to establish a T1D mouse model. Besides, CON mice were administered an equivalent volume of citrate buffer. At 3 h after STZ injection, all the mice were given a normal diet. Subsequently, blood glucose levels (BGL) were measured from tail vein blood after 3 d of STZ injection via a handheld glucometer (ACCU-CHEK Active, Roche, Mannheim, Germany). The mice with BGL exceeding 11.1 mmol/L were considered as T1D mice.

### 4.4. Establishment of type 2 diabetic (T2D) mouse model

The T2D mouse model was generated through a combination of a high-fat diet (HFD) and STZ administration (*n* = 8). Briefly, the mice were maintained on an HFD (D12492, Research Diets; 60 kcal% fat) for 4 weeks, followed by intraperitoneal injection of STZ dissolved in citrate buffer at 60 mg/kg once daily for 3 consecutive days. The CON mice were fed a normal diet and received an equal volume of citrate buffer by intraperitoneal injection (*n* = 8). Three days after the final STZ injection, blood glucose levels (BGLs) were determined from tail vein blood with a handheld glucometer (ACCU-CHEK Active, Roche, Mannheim, Germany). Mice with BGLs above 11.1 mmol/L were classified as T2D mice. Besides, the levels of fasting glucose and insulin were measured in the serum of the mice using ELISA kits (Wuhan Fine Biotech Co., Ltd., Wuhan, China), and the HOMA-IR value was calculated by (fasting glucose × fasting insulin)/22.5. After model establishment, T2D mice remained on the HFD until the end of the experiment.

### 4.5. Cell culture and cell counting kit-8 (CCK8) assay

SH-SY5Y cells were purchased from Shanghai FUHENG Biotechnology Co., Ltd. (Shanghai, China) and maintained in DMEM/F12 medium at a 1:1 ratio (BL305A, Biosharp, Beijing, China) containing 10% fetal bovine serum (FBS) and 1% penicillin‒streptomycin. The cells were cultured at 37 °C in a humidified incubator with 5% CO₂ (Thermo Fisher Scientific). Cell viability was assessed via the CCK8 assay (GK10001, GlpBio, Shanghai, China) according to the manufacturer’s protocols. To examine the impact of BCAAs on cell viability, SH-SY5Y cells were exposed to varying concentrations of leucine, valine, or isoleucine for 48 h, followed by incubation with CCK8 reagent at 37 °C for 1 h. To evaluate the synergistic effect of leucine and glucose on the cell viability, SH-SY5Y cells were treated with high glucose (33  mM) and different concentrations of leucine (0–75 mM) for 48 h, and then incubated with CCK8 reagent at 37 °C for 1 h. In addition, SH-SY5Y cells were administrated with high glucose, leucine or high glucose plus leucine for 6, 12, 24 and 36 h, and incubated with CCK8 reagent for 1 h at 37 °C. Finally, the absorbance at 450 nm was measured using a Thermo Fisher Scientific Microplate Reader (Wilmington, USA) to quantify the cell viability.

### 4.6. Fecal microbiota transplantation (FMT)

After 11 weeks of diabetic progression, a portion of T1D mice was randomly assigned to receive FMT (*n* = 8). Before FMT, the recipient mice underwent a 7-day microbiota-depletion period. During this period, the mice were orally gavaged with an antibiotic (ABX) cocktail containing vancomycin (50 mg/kg), neomycin (100 mg/kg), metronidazole (100 mg/kg), and amphotericin B (1 mg/kg), while ampicillin (1 g/L) was provided in the drinking water. Fresh feces were then obtained from CON mice and placed into sterile 1.5 mL centrifuge tubes under an anaerobic condition by using AnaeroPack (C-01, Mitsubishi, Tokyo, Japan) to minimize oxygen exposure. The PBS buffer was sonicated to reduce its oxygen content. The samples were suspended in pretreated PBS at a ratio of 1:7.5 (w/v), vortexed for 30 s, and centrifuged at 1000g for 2 min. The supernatant was collected as the fecal microbiota suspension. The recipient mice were administered 100  μL of this suspension by oral gavage once per day for 14 consecutive days.

### 4.7. Morris water maze test

To evaluate spatial learning and memory, the Morris water maze (MWM) test was performed as previously reported.^14^ Briefly, the experiment was conducted in a circular pool measuring 110  cm in diameter and 30  cm in height, which was filled with opaque water dyed with non-toxic white pigment at room temperature. A circular escape platform with a diameter of 7 cm was placed in the target quadrant and positioned 1 cm beneath the water surface. The MWM procedure included training and testing periods. During the training phase, the mice were given 1 min to locate the escape platform; if unsuccessful, the operator guided them to the platform. This procedure was repeated for 4 consecutive days with 4 training trials per day. On day 5, the escape platform was removed, and the trained mice were allowed 1 min of free exploration. The swimming paths were captured using an overhead video camera and analyzed with a computerized tracking system and “Viewer 2” software (Biobserve GmbH, Bonn, Germany). Behavioral indices were then quantified, including escape latency during the training period, as well as platform frequency and the proportions of swimming time and swimming distance spent in the original platform region during the probe trial.

### 4.8. *In vitro* culture of *Akkermansia muciniphila* (AKK)

AKK bacteria (ATCC BAA-835) were obtained from the American Type Culture Collection and grown in a brain heart infusion medium (BHI, CM1135B, Oxoid, UK) supplemented with mucin (S12066, Yuanye Bio-Technology, Shanghai, China) and L-cysteine (A600132-0100, Sangon Biotech, Shanghai, China) under a strictly anaerobic condition (5% H_2_, 5% CO_2_, and 90% N_2_) at 37 °C. The PBS buffer was sonicated to reduce its oxygen content. After 2 d, the AKK bacteria were harvested by centrifuging at 7000g for 5 min, resuspended in sterile pretreated PBS (pH = 7.4), and administered immediately after preparation. During the gavage procedures, the bacterial suspension was maintained in an anaerobic pouch (AnaeroPack, C-01, Mitsubishi, Tokyo, Japan). The viability of AKK bacteria was confirmed by anaerobic CFU enumeration prior to gavage. In this study, T1D mice (*n* = 7) were orally gavaged with either 0.2  mL sterile PBS or AKK suspension containing approximately 1 × 10^8^ CFU every other day for 12 weeks.

### 4.9. Quantification of AKK bacteria

Total genomic DNA was isolated from fecal samples in patients and mice using a Stool DNA kit (DP328-02, TIANGEN BlOTECH, Beijing, China) according to the manufacturer's instructions. The concentration of the extracted DNA was determined using a NanoDrop One spectrophotometer (Thermo Fisher Scientific, Waltham, MA, USA). Next, all bacterial DNA samples were diluted to the same concentration with Tris-Borate (TB) buffer. The quantitative detection of AKK bacteria in fecal samples was then measured by using SYBR Green PCR Master Mix (1725124, Bio-Rad, CA, USA) on a CFX384 Touch Real-Time PCR Detection System (Bio-Rad Laboratories, CA, USA). The relative abundance of AKK bacteria was calculated by the ΔΔCT method with universal 16S rRNA as the internal reference. The primer sequences for the universal 16S and AKK bacteria are provided in Table S2.

### 4.10. Bacterial load assay

To evaluate the effect of ABX treatment, we determined the bacterial load in the intestinal contents of the mice. Briefly, the intestinal contents were diluted by 10-fold serial dilution in sterile PBS, and 100  μL from each dilution was plated on Columbia blood agar plates, followed by incubation at 37 °C for 24  h under aerobic and anaerobic conditions, respectively. Finally, CFUs were counted and the results were expressed as CFU/g intestinal contents.

### 4.11. 16S rRNA sequencing analysis

Total genomic DNA was extracted from fecal specimens using the TIANamp Stool DNA Kit according to the manufacturer’s instructions (TianGen, China). The quality and quantity of the extracted DNA were evaluated by agarose gel electrophoresis and a NanoDrop NC-2000 spectrophotometer (Thermo Fisher Scientific, Waltham, MA, USA). The V3-V4 hypervariable region of the bacterial 16S rRNA gene was amplified using the 338F/806R primer pair. The PCR amplicons were purified with Vazyme VAHTS™ DNA Clean Beads (Vazyme, Nanjing, China) and quantified using the Quant-iT PicoGreen dsDNA Assay Kit (Invitrogen, Carlsbad, CA, USA) with a BioTek FLx800 microplate reader. Sequencing libraries were then constructed using the NovaSeq 6000 SP Reagent Kit with 500 cycles and sequenced on the Illumina NovaSeq platform (Illumina, San Diego, CA, USA).

The raw sequencing data were subjected to standard bioinformatic processing. Paired-end reads were assembled using FLASH (v1.2.7), and low-quality sequences were removed with QIIME2 to obtain high-quality clean tags.^59^ Chimeric reads were identified and excluded using the UCHIME algorithm. The remaining valid sequences were clustered into operational taxonomic units (OTUs) at a 97% similarity threshold using the UPARSE pipeline.^60^ Taxonomic assignment of OTUs was conducted against the SILVA database using the Mothur algorithm, with a confidence cutoff of 80%.

### 4.12. Determination of amino acid profiles

In this study, the serum levels of 22 amino acids in mice were determined by using LC–MS-based targeted metabolomics. For sample preparation, 50 μL of serum was mixed with 150 μL of acetonitrile and subjected to ultrasonic extraction for 5  min. The mixture was subsequently centrifuged at 13,000g for 5 min at 4 °C, after which 40 μL of the supernatant was collected and evaporated to dryness under nitrogen. The dried extracts were reconstituted in 160  μL of 50% acetonitrile and centrifuged at 13,000 g for 5 min at 4 °C. The resulting supernatant was collected for amino acid quantification. Serum amino acid profiling was performed using an ExionLC AD system coupled to an AB SCIEX QTRAP 6500 + mass spectrometer (AB Sciex, USA). Chromatographic separation was achieved on a Waters BEH Amide column (100 × 2.1 mm, 1.7 μm) maintained at 35 °C, with an injection volume of 2  μL. The mobile phase consisted of solvent A (20 mM ammonium formate, 0.4% formic acid in 95% acetonitrile) and solvent B (20 mM ammonium formate, 0.4% formic acid in 5% acetonitrile aqueous solution). The gradient elution procedure was set as follows: 0–1.0 min, 0%–10% B; 1.0–2.6 min, 10%–15% B; 2.6–3.5 min, 15%–30% B; 3.5–4.0 min, 30% B; 4.0–4.1 min, 30%–0% B; and 4.1–6 min, hold at 0% B until the end of separation. LC‒MS analysis was conducted with an electrospray ionization (ESI) source operating in positive mode. The main parameters were set as follows: source temperature, 550 °C; curtain gas, 35 psi; ion sources Gas1 and Gas2, 70 psi; and ion-spray voltage floating, 5500 V. All 22 amino acid standards were purchased from Sigma-Aldrich, as shown in Table S3. The calibration curves showed good linearity over the concentration range of 0.02–200 μmol/L, with correlation coefficients greater than 0.99.

### 4.13. Targeted detection of leucine and STZ using UPLC-MS

Approximately 20 mg of intestinal contents or fecal material was weighed and extracted with 500 μL of pre-cooled 50% acetonitrile. For the serum and hippocampal samples, 20 μL of serum or 20 mg of tissue was placed into a centrifuge tube and mixed with 200  μL of methanol/acetonitrile extraction solvent at a ratio of 1:1 (v/v). All the samples were homogenized for 2 min using a tissue homogenizer (Jingxin, Shanghai, China), kept at −20 °C for 30 min, and subsequently centrifuged at 15,000g for 15 min at 4 °C. The resulting supernatants were transferred into new tubes, dried by centrifugal evaporation, and stored at −80 °C until further analysis. Before LC‒MS detection, the dried residues were redissolved in 50% acetonitrile and centrifuged at 15,000g for 20 min at 4 °C. The final supernatants were then loaded into autosampler vials for analysis.

The quantifications of leucine and STZ were performed using a Waters ACQUITY UPLC H-Class Plus system coupled to a XEVO TQ-S micro mass spectrometer (Waters, Milford, MA, USA). Chromatographic separation was achieved on a UPLC BEH HILIC column (2.1 mm × 100 mm, 1.7 μm) maintained at 40 °C, with a flow rate of 0.4  mL/min. The mobile phase included solvent A (0.1% formic acid in water) and solvent B (acetonitrile), and the gradient elution procedure was set as follows: 0–1.5 min, 90% B; 1.5–2.6 min, 15% B; 2.6–2.7 min, 15% B; and 2.7–4 min, 90% B. The eluted analytes were detected using electrospray ionization in multiple-reaction monitoring mode. The chromatographic condition for STZ were as follows: column temperature, 40 °C; flow rate, 0.4 mL/min; and injection volume, 5 μL. The mobile phase consisted of solvent A (0.1% formic acid aqueous solution) and solvent B (0.1% formic acid acetonitrile solution). The gradient elution program was optimized as follows: 0 min, 90% solvent B; 1.5 min, 15% solvent B; 1.5–2.6 min, maintained at 15% solvent B; 2.7 min, returned to 90% solvent B; and the total running time was 4.0 min. The concentration of streptozotocin in all the samples was quantitatively determined based on the established standard curve. The main MS parameters included: ion source temperature, 150 °C; desolvation gas temperature, 500 °C; cone gas flow rate, 50 L/h; desolvation gas flow rate, 1000 L/h; and argon gas flow rate, 0.15 mL/min. The standard curves of leucine and STZ exhibited the linear range of 40–1200  ng/mL with an R^2^ > 0.99.

### 4.14. Histopathological examination

Mice (*n* = 6–9 per group) were randomly selected, anesthetized with isoflurane, and then euthanized via cervical dislocation. The brain tissue was quickly excised and fixed in 4% paraformaldehyde (BL539A, Biosharp, China) for 24 h. After graded dehydration, the tissues were embedded in paraffin and sectioned into 5-μm-thick slices for subsequent staining procedures. In this study, histopathological staining procedures were kept consistent across different groups within the same experimental batch, including antibody concentration, antibody incubation time, chromogenic development time, and image acquisition parameters. For histopathological examination, hippocampal paraffin sections were deparaffinized and stained with hematoxylin (ZLI-9610, ZSGB-BIO, Beijing, China) and eosin (ZLI-9613, ZSGB-BIO, Beijing, China). Nissl staining was conducted by incubating the sections with Nissl staining solution (C0117, Beyotime, Shanghai, China) at 37 °C for 3 h. Finally, hippocampal morphology was examined and imaged using a light microscope (Nikon, Tokyo, Japan).

For immunohistochemical staining, hippocampal sections were incubated at 65 °C for 8–10 h. After deparaffination and rehydration, the sections were washed with PBS for 15 min. Heat-induced antigen retrieval was performed by boiling the sections in 10 mM sodium citrate buffer (pH 6.0) at 100 °C for 5 min. The sections were then washed with PBS and treated with 3% H₂O₂ (PO100A, Beyotime, Shanghai, China) for 10 min to quench endogenous peroxidase activity. After blocking with 5% BSA for 30 min, the sections were incubated overnight at 4 °C with primary antibodies against NeuN (D4G4O) XP (1:500, #24307, Cell Signaling Technology) and TOM20 (1:200, AF5206, Affinity). After PBS washing, the sections were incubated with an HRP-conjugated goat anti-rabbit secondary antibody (1:200, SA00001-2, Proteintech) at 37 °C for 1 h. The signals were developed using a DAB chromogenic kit (ZLI-9018, ZSGB-BIO, Beijing, China). The sections were counterstained with hematoxylin for 1 min and mounted with neutral resin. Finally, the images were examined under a light microscope (Nikon, Tokyo, Japan) and analyzed using ImageJ software.

For immunofluorescence analysis, SH-SY5Y cells were plated onto coverslips and fixed with 4% paraformaldehyde for 30 min. The tissue sections and cells were then blocked with 5% BSA at 37 °C for 30  min, followed by overnight incubation at 4 °C with primary antibodies against LC3B (1:100, AF4650, Affinity), TOM20 (1:100, ab289670, Abcam), OPTN (1:200, 60293-1-Ig, Proteintech), and TBK1 (1:100, DF7026, Affinity). After PBS washing, the samples were incubated with the corresponding secondary antibodies at 37 °C for 1 h, including Alexa Fluor 488-conjugated anti-mouse IgG (1:100, HA1125, HUABIO), DyLight 488-conjugated anti-rat IgG (1:100, E0322240-01, Earthox), and Alexa Fluor 555-conjugated anti-rabbit IgG (1:100, AB150078, Abcam). Cell nuclei were counterstained with DAPI (C0065; Solarbio, Beijing, China) for 15 min. Images were captured using a Nikon C2si confocal microscope (Nikon, Tokyo, Japan).

### 4.15. RNA isolation and RT-qPCR analysis

Total RNA was extracted from tissue and cultured cell samples using TRIzol reagent (Cat#9109; TaKaRa, Beijing, China), and its concentration was measured with a NanoDrop spectrophotometer (Thermo Fisher Scientific, Beverly, MA, USA). The purified RNA was subsequently converted into complementary DNA using HiScript III RT SuperMix (R323-01; Vazyme, Nanjing, China). Quantitative real-time PCR was performed with SYBR Green PCR Master Mix (1725124; Bio-Rad, CA, USA) on a CFX384 Touch Real-Time PCR Detection System (Bio-Rad Laboratories, CA, USA). Gene expression levels were normalized to *β*-actin and calculated using the ΔΔCT method. All primer sequences were synthesized by Tsingke Biotechnology (Beijing, China) and are provided in Table S2.

### 4.16. Western blot (WB) analysis

Total proteins in tissue and cell samples were extracted using the lysis buffer (AR0101; AR0103-100; BOSTER, Wuhan, China) with protease inhibitors (1  mM, p1260; APPLYGEN, Beijing, China) and phosphatase inhibitors (ST506-2, Beyotime, Shanghai, China). Afterward, samples were maintained on ice for 30  min, followed by homogenization and sonication. After centrifugation at 14,000g for 15  min at 4 °C, the supernatants were collected, and protein concentrations were determined using the Coomassie brilliant blue assay. Equal amounts of protein from each sample were resolved by 10% SDS‒PAGE and then transferred onto PVDF membranes (ISEQ00010, Millipore, Germany). The membranes were blocked by 5% non-fat milk (1172GR500, Biofroxx, Germany) for 1 h at room temperature and incubated overnight at 4 °C with specific primary antibodies, including *β*-actin (1:10000, 66009-1-Ig, Proteintech), LC3B (1:1000, AF4650, Affinity Biosciences), pan-Phospho-Serine Rabbit pAb (*p*-Ser, 1:1000, AP0932, Abclonal), Ubiquitin (Ub, 1:1000, YP-mAb-18538, UpingBio), phospho-TBK1 (*p*-TBK1, 1:1000, AP0847, Abclonal), OPTN (1:1000, 60293-1-Ig, Proteintech), and TBK1 (1:1000, DF7026, Affinity Biosciences). After three washes with TBST, the membranes were incubated with HRP-conjugated secondary antibodies for 1.5 h at room temperature. The protein bands were subsequently visualized using a ChemiDoc XRS +  imaging system (Bio-Rad, CA, USA), and the band densities were analyzed with ImageJ software (v1.47, Bethesda, MD, USA).

### 4.17. Transfection with siRNA

Small interfering RNA (siRNA) was employed to silence TBK1 expression in SH-SY5Y cells. Briefly, cells were plated in 6-well plates and cultured until they reached approximately 70%–80% confluence. The cells were then transfected with either TBK1-specific siRNA or negative control siRNA (si-NC) using Lipofectamine 3000 (Invitrogen, USA) according to the manufacturer’s protocol. For transfection, Lipofectamine 3000 and siRNA were separately diluted in Opti-MEM medium, gently combined, and incubated at room temperature for 15 min before adding to the cells. The transfection efficiency and TBK1 knockdown were confirmed by RT-PCR and Western blotting. All siRNAs were synthesized by General Biol Co., Ltd. (Anhui, China), and the specific sequences are listed in Table S4.

### 4.18. Measurement of mitochondrial membrane potential by JC-1 staining

The mitochondrial membrane potential in SH-SY5Y cells was determined using the JC-1 fluorescent probe with the manufacturer’s instructions (C2006, Beyotime, Shanghai, China). Briefly, SH-SY5Y cells were plated in confocal dishes at a density of 1 × 10^6^ cells per well. After the designated treatments, the cells were rinsed three times with PBS and stained with 5  μg/mL JC-1 working solution at 37 °C for 20  min in a cell culture incubator. After staining, the cells were washed twice with PBS, suspended in 1 mL of incubation buffer, and promptly observed under a Nikon C2si confocal microscope (Nikon, Tokyo, Japan). Green fluorescence represents the monomeric form of JC-1 and reflects mitochondrial depolarization, whereas red fluorescence corresponds to JC-1 aggregates and indicates intact mitochondrial membrane potential. The fluorescence signals were quantified using ImageJ software (version 1.47; Bethesda, MD, USA).

### 4.19. Intracellular reactive oxygen species (ROS) assays

Intracellular ROS levels in SH-SY5Y cells were measured using the DCFH-DA fluorescent probe. In brief, cells were seeded into 6-well plates and loaded with 10  μM DCFH-DA (S0033S, Beyotime, Shanghai, China) at 37 °C for 60  min. After incubation, the cells were rinsed three times with PBS to eliminate unbound dye. ROS-associated fluorescence was captured using a Nikon C2si confocal microscope (Nikon, Tokyo, Japan), and the signal intensity was analyzed with ImageJ software (version 1.47; Bethesda, MD, USA).

### 4.20. Determination of ATP levels

The ATP level was determined with ATP assay kit (S0026; Beyotime, Shanghai, China) according to the manufacturer's protocols. The cells were thoroughly lysed with ATP lysis buffer at 4 °C, and the lysates were centrifuged to collect the supernatant. Afterward, the ATP working reagent was dispensed into a 96-well plate and added with 50  μL of the supernatant. After a 5-min incubation, the intracellular ATP levels were quantified using a microplate reader (Synergy NEO2, BioTek, VT, USA).

### 4.21. Screening of the direct protein targets of leucine

In this study, direct protein targets of leucine were screened using a combined strategy involving drug affinity responsive target stability (DARTS) and Astral DIA-based quantitative proteomics.^61^ SH-SY5Y cells were first rinsed with PBS and lysed on ice for 15 min in lysis buffer (AR0103-100, BOSTER, Wuhan, China) supplemented with 1% phosphatase and protease inhibitors. The cell lysates were transferred into microcentrifuge tubes and centrifuged at 14,000g for 15 min at 4 °C. The supernatants were collected and incubated with either 10 mM leucine or a vehicle control at room temperature for 2 h. Subsequently, the protein mixtures were digested with pronase (10165921001, Roche, Basel, Switzerland) for 15 min. An equal volume of loading buffer was then added, and the samples were thoroughly mixed and heated at 100 °C for 10 min. Proteins were separated by SDS–PAGE and visualized using Coomassie brilliant blue staining solution (PT0018; Leagene, Beijing, China).

The target protein bands were carefully excised from the gel using a scalpel and placed into centrifuge tubes. After adding 1 mL of distilled water, the gel pieces were shaken at 1,000  rpm for 2 min. The gel pieces were incubated in 800  μL of destaining solution (100  mmol/L NH₄HCO₃/30% ACN) at 37 °C with shaking at 1,000  rpm for 5  min, and this step was repeated until the gel became clear. Then, 360 μL of 100 mmol/L NH₄HCO₃ and 40 μL of 100 mmol/L DTT were added, and the samples were incubated at 56 °C for 30 min. After removing the supernatant, 280  μL of 100 mmol/L NH₄HCO₃ and 120  μL of 200 mmol/L IAA were added and incubated in the dark for 30  min. The supernatant was then removed again, and 2 μL of trypsin was added to each tube. In-gel digestion was performed at 37 °C for 20 h overnight. The peptide solutions were transferred into new tubes, mixed with 100  μL of 60% acetonitrile containing 0.1% TFA, sonicated for 15 min, and lyophilized to dryness. The peptides were further purified using C18 desalting tips. The tips were first activated with 100% acetonitrile and equilibrated with 0.1% formic acid. Peptide samples were loaded onto the tips, washed with 0.1% formic acid, and finally eluted with 70% acetonitrile containing 0.1% formic acid. The eluates were collected and dried in a vacuum concentrator.

Peptide separation was performed on a nanoflow Vanquish Neo UHPLC system fitted with an EASY-Spray C18 column (150 μm × 15  cm) at 55 °C. The mobile phases included solvent A, 0.1% formic acid in water, and solvent B, 0.1% formic acid in 80% acetonitrile. Peptides were eluted using the following gradient program: 4%–8% B from 0.0 to 0.1 min, 8%–28% B from 0.1 to 5.1 min, 28%–43% B from 5.1 to 6.9 min, 43%–99% B from 6.9 to 7.3 min, and 99% B from 7.3 to 8.0 min. The separated peptides were then detected on an Astral high-resolution mass spectrometer (Thermo Scientific) operated in data-independent acquisition (DIA) mode. MS1 spectra were acquired over an m/z range of 380–980 at a resolution of 240,000 at m/z 200, with the normalized AGC target and maximum injection time set to 500% and 5 ms, respectively. MS2 spectra were acquired in DIA mode using 300 scan windows with a width of 2 m/z, an HCD collision energy of 25 eV, a normalized AGC target of 500%, and a maximum injection time of 3 ms. The DIA data analysis was processed with DIA-NN software (v1.9.2) based on the reviewed UniProt human database. The search parameters were configured as follows: trypsin as the digestion enzyme, one missed cleavage allowed, carbamidomethylation as a fixed modification, and oxidation and protein *N*-terminal acetylation as variable modifications. Only proteins identified with 99% confidence and a false discovery rate (FDR) of ≤ 1% were retained for further analysis.

### 4.22. Drug affinity responsive target stability (DARTS)

In this study, DARTS was used to validate the interaction between leucine and TBK1.^62^ Briefly, SH-SY5Y cells were washed with PBS and lysed on ice for 15 min in lysis buffer (AR0103-100, BOSTER, Wuhan, China) supplemented with 1% phosphatase (P1260, Applygen, Beijing, China) and protease (ST506-2, Beyotime Biotechnology, Shanghai, China) inhibitors. The lysates were then transferred into microcentrifuge tubes and centrifuged at 14,000g for 15 min at 4 °C. The supernatants were collected and then incubated with 10 mM leucine or vehicle control at room temperature for 2 h. After incubation, the samples were subjected to pronase digestion (10165921001, Roche, Basel, Switzerland) at a 1:800 ratio (w/w) for 15 min at room temperature. Subsequently, the reaction was terminated by the addition of an equal volume of loading buffer, and the mixtures were thoroughly mixed and boiled for 10 min. Finally, the treated samples were analyzed by Western blotting to evaluate changes in TBK1 protein abundance.

### 4.23. Cellular thermal shift assay (CETSA)

Furthermore, CETSA was employed to further verify the interaction between leucine and TBK1.^63^ SH-SY5Y cells were washed with PBS and lysed on ice in the lysis buffer (AR0103-100, BOSTER, Wuhan, China) containing 1% phosphatase and protease inhibitors for 15 min. The lysates were transferred into microcentrifuge tubes and centrifuged at 14,000g for 15 min at 4 °C. The supernatants were collected and subsequently incubated with either 10 mM leucine or a vehicle control at room temperature for 2 h. The samples of each group were divided into 7 tubes equally, boiled at different temperatures, including 42, 47, 52, 57, 62, 67, and 72 °C, for 5  min, and then cooled at room temperature for 3  min. The samples were then centrifuged at 13,000g for 15 min, and the supernatants were mixed with loading buffer. Finally, TBK1 protein levels were examined by Western blotting.

### 4.24. Adeno-associated virus (AAV)-mediated knockdown of TBK1

Adeno-associated virus (AAV) vectors were used to achieve neuron-specific TBK1-knockdown in mice. AAV-PHP.eB-hsyn-shTBK1 (shTBK1) and AAV-PHP.eB-hsyn-shNC (shNC) were constructed from the General Biol Co., Ltd. (Anhui, China). In this study, T1D mice received tail vein injections of 200  μL of shTBK1 solution with a viral titer of 1.5 × 10^12^ at 0 and 6 weeks after STZ administration, while another group of T1D mice was injected with the same volume of shNC solution as negative controls. TBK1 expression levels were subsequently evaluated by immunofluorescence staining and RT-qPCR.

### 4.25. Co-immunoprecipitation (Co-IP) assay

The Co-IP assay was carried out to validate protein‒protein interactions in this study. Briefly, proteins in animal tissue and cell samples were extracted using 0.3  mL of NP40 lysis buffer (P0013F; Beyotime Technology, Shanghai, China) supplemented with 1  mM PMSF (ST506; Beyotime Technology, Shanghai, China). A portion of each lysate was retained as the input control. After centrifugation, the protein extracts were incubated overnight at 4 °C with 1 μg of anti-OPTN antibody (60293-1-Ig, Proteintech) or control IgG under gentle agitation. Subsequently, 50  μL of protein A/G agarose beads (P2055, Beyotime Technology, Shanghai, China) was added, and the mixtures were further incubated at 4 °C for 3 h. The samples were then centrifuged at 1000 × g for 3 min, and the supernatants were discarded to collect the immunocomplexes. The immunocomplexes were washed five times with PBS, resuspended in SDS loading buffer, and boiled at 100 °C for 10 min. Finally, the prepared samples were subjected to Western blot analysis.

### 4.26. Statistical analysis

In this study, all the mice were randomly allocated throughout the experimental workflow, including group assignment, intervention administration, sample collection, and data processing. Before multivariate statistical analysis, omics data were subjected to log transformation followed by Pareto scaling. Principal component analysis (PCA) was performed using SIMCA-P+  software (v12.0, Umetrics AB, Umeå, Sweden) to examine the changes in amino acid profiles across groups. Gut microbiota beta diversity was analyzed by principal coordinate analysis (PCoA) based on Bray-Curtis dissimilarity using R software (v2.15.3). Statistical differences among different groups were analyzed with SPSS 22.0 software (SPSS Inc., Chicago, IL, USA). Differences between two groups were examined using a two-tailed unpaired Student’s *t*-test, whereas comparisons with multiple groups were performed by one-way or two-way ANOVA followed by Dunnett’s or Sidak post hoc test. The significance of differences between various groups over time was evaluated by two-way repeated measures ANOVA. A *p*-value of less than 0.05 was considered statistically significant. Volcano plots were generated through plotting log₂(fold change) against –log₁₀(*p* value) in R software (v2.15.3). Correlations among variables were determined using Spearman correlation analysis in SPSS 22.0 software, and correlation networks were visualized with Cytoscape software (v3.10.3).

## Supplementary Material

Supplementary Material Supplemental Information_R1.docx

## Data Availability

All data used in this study are presented in the main text and supplementary materials. Omics data have been made publicly available in Figshare: Metabolomics (DOI: 10.6084/m9.figshare.30739295); Microbiomics (DOI: 10.6084/m9.figshare.30738932); Proteomics (DOI: 10.6084/m9.figshare.30744734).
