# Supplementary material for: Akkermansia muciniphila protects diabetes-associated cognitive decline by enhancing leucine catabolism and suppressing neuronal excessive mitophagy
Source: Gut Microbes. 2026 Sep 30;18(1):2739666. doi: 10.1080/19490976.2026.2739666 (PMC13641599; doi:10.1080/19490976.2026.2739666)
Supplement: Supplementary file 1 [file KGMI_A_2739666_SM6064.docx]

*Akkermansia muciniphila* protects diabetes-associated cognitive decline by enhancing leucine catabolism and suppressing neuronal excessive mitophagy

Xianxi Ye^1^, Qingpeng Cai^1^, Yulong Li^1^, Mingjie You^1^, Junli Chen^1^, Yangjie Pan^1^, Ming Zhu^1^, Zhuofan Lu^1^, Siyuan Chen^2^, Yan Lin^3^, Weiru Zhang^1^, Xuelian Liu^1^, Zhen Wang^4^, Guangliang Hong^5,6,^ Wenjun Wu^3,*^, & Hong Zheng^1,*^

^1^State Key Laboratory of Macromolecular Drugs and Large-scale Preparation, School of Pharmaceutical Sciences, Wenzhou Medical University, Wenzhou 325035, China

^2^Forensic Medicine Center, Wenzhou Medical University, Wenzhou 325027, China

^3^Department of Endocrinology and Metabolism, The First Affiliated Hospital of Wenzhou Medical University, Wenzhou 325000, China

^4^Department of Neurology, The First Affiliated Hospital of Wenzhou Medical University, Wenzhou 325000, China

^5^Department of Emergency Medicine, The First Affiliated Hospital of Wenzhou Medical University, Wenzhou 325000, China

^6^Department of Emergency Medicine, The Quzhou Affiliated Hospital of Wenzhou Medical University, Quzhou People’s Hospital, Quzhou 324000, China

*Corresponding author:

Wenjun Wu ([wwju127@wmu.edu.cn](mailto:wwju127@wmu.edu.cn));

Hong Zheng ([123zhenghong321@163.com](mailto:123zhenghong321@163.com)).

**Table S1.** Clinical characteristics of volunteers recruited in this study.

| Item | Cohort 1 | | | | Cohort 2 |
| --- | --- | --- | --- | --- | --- |
|  | Health^a^ (n=25) T1D^b^ (n=19) T2D^c^ (n=25) P value | | | | T2D (n=145) |
| Age (years) | 54.96 ± 10.60^f^ | 59.11 ± 5.95 | 56.52 ± 7.57 | 0.278 | 56.34 ± 11.90 |
| BMI^d^ (kg/m^2^) | 23.04 ± 3.07 | 20.24 ± 4.48 | 23.70 ± 3.53 | 0.008 | 24.04 ± 3.22 |
| FBG^e^ (mmol/L) | 5.37 ± 0.60 | 8.82 ± 4.05 | 8.29 ± 3.73 | 0.001 | 8.09 ± 2.94 |
| HbA1c (%) | 5.80 ± 0.36 | 10.00 ± 3.38 | 8.94 ±2.86 | ＜0.001 | 9.58 ± 2.24 |
| Diabetes duration (years) | 0.00 ± 0.00 | 11.69 ± 10.20 | 10.74 ± 5.87 | ＜0.001 | 10.88 ± 6.78 |
| Sex (male/female) | 12/13^g^ | 8/11 | 11/14 | 0.920 | 90/55 |
| Smoking (%) | 3 (12%) | 3 (15.79%) | 4 (16%) | ＞0.999 | 43 (29.66%) |
| Drinking (%) | 3 (12%) | 5 (26.32%) | 4 (16%) | 0.443 | 36 (24.83%) |
| Hypertension (%) | 7 (28%) | 4 (21.05%) | 11 (44%) | 0.236 | 74 (51.03%) |

^a^Healthy controls; ^b^Patients with type 1 diabetes; ^c^Patients with type 2 diabetes; ^d^Body mass index; ^e^Fasting blood glucose level; ^f^The normally distributed continuous data satisfying the homogeneity of variance were presented as mean ± standard deviation (SD) and compared using one-way analysis of variance (ANOVA); ^g^The categorical data were presented as the number (percentage) and compared using the Pearson χ2 test and Fisher’s exact test.

| **Table S2.** Primer sequences used for real-time qPCR analysis. | | | |
| --- | --- | --- | --- |
| Gene | Species | Sequence (Forward) | Sequence (Reverse) |
| ACTIN | Mouse | GTGACGTTGACATCCGTAAAGA | GCCGGACTCATCGTACTCC |
| SYP | Mouse | AAGTATTGTGGTTTGGAG | GTAGATTCTGTCTTCTGATT |
| ARC | Mouse | GACCTGACATCCTGGCACC | TTCTCCTGGCTCTGTAGGCT |
| PSD95 | Mouse | CCAGGCTACTATTCATAAG | ATCATCGCTCAGTATAAAC |
| EGR-1 | Mouse | ACTCTGCTGTGACATTAGG | AACGGAACAACACTCTGA |
| C-FOS | Mouse | TTCTCATAGCACTAACTAATCT | CAGGAACACAGTAGGTATT |
| BCAT | Mouse | CAGCCACACTAGGACAGGTCT | CAGCCTTGTTATTCCACTCCAC |
| BCKDH | Mouse | CTCCTGTTGGGACGATCTGG | CATTGGGCTGGATGAACTCAA |
| Atp2a1 | Mouse | TGTTTGTCCTATTTCGGGGTG | AATCCGCACAAGCAGGTCTTC |
| Slc25a4 | Mouse | AAGGTGAAGAGCATCATAACCCT | TCACGCCTTTCATAACACATTCC |
| Atp1a1 | Mouse | AAGGTGAAGAGCATCATAACCCT | TCACGCCTTTCATAACACATTCC |
| Oxsm | Mouse | GCTGCTTATGTACCAAGAGGTC | CCAGCCGGAGTCTTTCAGG |
| Cnp | Mouse | GACTTCTCCGAGGCGTACAAG | CCGCTCGTGGTTGGTATCA |
| mt-Atp6 | Mouse | CTGGTGGCGGGTGCTTTAG | GCTACGTCTGGGATTCGATCT |
| Rpl13 | Mouse | AGCCGGAATGGCATGATACTG | ATCTCACTGTAGGGCACCTCA |
| TBK1 | Mouse | ACTGGTGATCTCTATGCTGTCA | TTCTGGAAGTCCATACGCATTG |
| ACTIN | Human | GAAGATCAAGATCATTGCTCCTC | ATCCACATCTGCTGGAAGG |
| Atp2a1 | Human | AAACCACGGAGGAATGTTTGG | AGCTCATTGAGGCCGTATTTC |
| Slc25a4 | Human | ATCACGCTTGGAGCTTCCTAA | TGCTTCTCAGCACTGATCTGT |
| Atp1a1 | Human | ACAGACTTGAGCCGGGGATTA | TCCATTCAGGAGTAGTGGGAG |
| Oxsm | Human | CAATATCCAGATTGCATAGGCGA | CGATCCCAAACCAGGTGAGTT |
| Cnp | Human | AACAGAGGCTTCTCCCGAAAA | GTCTTGCACTCTAGCAGCGT |
| Mt-Atp6 | Human | GTCCTAACCGGGGAGTTATCA | AAAGAGAGGGTTGAGTTGGGC |
| Rpl13 | Human | GCCATCGTGGCTAAACAGGTA | GTTGGTGTTCATCCGCTTGC |
| TBK1 | Human | TGGGTGGAATGAATCATCTACGA | GCTGCACCAAAATCTGTGAGT |
| *A.muciniphila* | Bacterial | CCTTGCGGTTGGCTTCAGAT | CAGCACGTGAAGGTGGGGAC |
| 16S | Bacterial | ACTCCTACGGGAGGCAGCAGT | GTATTACCGCGGCTGCTGGCAC |

**Table S3.** Composition of amino acid profile in this study.

| Amino acid | Source | Identifier |
| --- | --- | --- |
| L-Alanine | Shanghai Yuanye Bio-Technology Co., Ltd. | B21911 |
| L-(+)-Arginine | Shanghai Yuanye Bio-Technology Co., Ltd. | B21920 |
| L-Asparagine Anhydrous | Shanghai Yuanye Bio-Technology Co., Ltd. | B21935 |
| L-Aspartic Acid | Shanghai Yuanye Bio-Technology Co., Ltd. | B21934 |
| L-Cysteine | Shanghai Yuanye Bio-Technology Co., Ltd. | B21909 |
| L-Glutamine | Shanghai Yuanye Bio-Technology Co., Ltd. | B21917 |
| L-Glutamic Acid | Shanghai Yuanye Bio-Technology Co., Ltd. | B21916 |
| Glycine | Shanghai Yuanye Bio-Technology Co., Ltd. | B21915 |
| L-Histidine | Shanghai Yuanye Bio-Technology Co., Ltd. | B21938 |
| L-Hydroxyproline | Shanghai Yuanye Bio-Technology Co., Ltd. | B21928 |
| L-Isoleucine | Shanghai Yuanye Bio-Technology Co., Ltd. | B21937 |
| L-Leucine | Shanghai Yuanye Bio-Technology Co., Ltd. | B21925 |
| L-(+)-Lysine | Shanghai Yuanye Bio-Technology Co., Ltd. | B21922 |
| L-Methionine | Shanghai Yuanye Bio-Technology Co., Ltd. | B21913 |
| L-Phenylalanine | Shanghai Yuanye Bio-Technology Co., Ltd. | B21910 |
| L-Proline | Shanghai Yuanye Bio-Technology Co., Ltd. | B21914 |
| L-Serine | Shanghai Yuanye Bio-Technology Co., Ltd. | B21932 |
| L-(-)-Threonine | Shanghai Yuanye Bio-Technology Co., Ltd. | B21933 |
| L-Tryptophan | Shanghai Yuanye Bio-Technology Co., Ltd. | B21930 |
| L-(-)-Tyrosine | Shanghai Yuanye Bio-Technology Co., Ltd. | B21924 |
| L-Valine | Shanghai Yuanye Bio-Technology Co., Ltd. | B21936 |
| L-Cystine | GlpBio | GA11210 |

**Table S4.** Specific siRNA sequences for different target genes.

| Gene name | Sense (5'-3') | Antisense (5'-3') |
| --- | --- | --- |
| si-TBK1 1# | GGAAUAAAGAAGUGAUGUATT | UACAUCACUUCUUUAUUCCTT |
| si-TBK1 2# | CCACAAAUUUGAUAAGCAATT | UUGCUUAUCAAAUUUGUGGTT |
| si-TBK1 3# | CCAAGGAGCUACUGCAAAUTT | AUUUGCAGUAGCUCCUUGGTT |
| si-NC | UUCUCCGAACGUGUCACGUTT | ACGUGACACGUUCGGAGAATT |

**Figure S1.** Cognitive decline occurs in the advanced stages of diabetes in mice. (a) Experimental procedure: In this study, type 1 diabetic (T1D) mice were established by intraperitoneal injection of streptozotocin (STZ) and their learning and memory abilities were evaluated after 11 weeks of diabetic progression via the Morris water maze (MWM) test. (b) The escape latency during a 4-day training period of the MWM test in normal control (CON) and T1D mice (n = 6-7). (c) The swimming trajectories of CON and T1D mice during the test period of the MWM test. (d-f) The platform frequence (d) and the percentages of swimming time (e) and distance (f) in the original platform area in CON and T1D mice during the test period of the MWM test (n = 6-7). Data were presented as means ± standard errors. The statistic difference between two groups was assessed by two-tailed unpaired student’s *t* test and *p*<0.05 was considered as a statistically significant difference. The significant of difference in the escape latency during a 4-day training period was evaluated by two-way repeated measure ANOVA: G*T, interaction effect of group and time; G, main effect of group; T, main effect of time. Significance level: *p<0.05; **p<0.01.

**Figure S2.** The effect of intraperitoneal streptozotocin (STZ) injection on neurons and cognitive functions in mice. (a, b) Representative immunofluorescence staining images of GLUT2 and the corresponding quantitative data in pancreas and hippocampus of mice (n = 5). Scale bar = 100 μm. (c) Experimental procedure: Serum samples were collected from the tail vein of mice before intraperitoneal STZ injection and at 5, 10, 15, 30, 60, and 120 min after injection, followed by measurement of STZ concentrations over time. (d, e) Time-course profiles of STZ concentrations in the (d) serum and (e) pancreas and hippocampus tissues of mice after intraperitoneal STZ injection (n = 5). (f) Changes in SH-SY5Y cell viability after 2 h exposure to different concentrations of STZ by the CCK8 test (n = 5). (g) Experimental procedure: Mice received an intraperitoneal STZ injection for five consecutive days, and their learning and memory abilities were assessed using the Morris water maze (MWM) test after 4 weeks. (h) The escape latency during a 4-day training period of the MWM test in normal control (CON) and type 1 diabetic (T1D) mice (n = 6). (i-k) The percentages of swimming time (i) and distance (j) in the original platform area and the platform frequence (k) in CON and T1D mice during the test period of the MWM test (n = 6). Data were presented as means ± standard errors. The statistic difference between two groups was assessed by two-tailed unpaired student’s *t* test and *p*<0.05 was considered as a statistically significant difference. The significant of difference in the escape latency during a 4-day training period was evaluated by two-way repeated measure ANOVA. Significance level: n.s., no significant difference; ***p<0.001.

**Figure S3.** Transplantation of fecal microbiota from healthy mice relieves neuronal injury in the hippocampus of diabetic mice. (a, b) Representative images of H&E (a) and Nissl (b) staining in the CA1, CA3 and DG regions of the hippocampus in normal control (CON) mice, T1D mice, and T1D mice receiving FMT from healthy donors. Scale bar = 100 μm.

**Figure S4.** The effects of branched-chain amino acids (BCAAs) on neuronal cell viability. (a-c) The changes of SH-SY5Y cell viabilities after treatment with different concentrations of leucine (a), valine (b) and isoleucine (c) under a normal glucose (NG, 5.5 mM) condition for 48 h (n = 5). (d) The changes of SH-SY5Y cell viabilities after treatment with different concentrations of leucine under a high glucose (HG, 33 mM) condition for 48 h (n = 5). Data were presented as means ± standard errors. The statistic difference between two groups was assessed by two-tailed unpaired student’s *t* test and *p*<0.05 was considered as a statistically significant difference. Pairwise comparisons were conducted between BCAAs treatment groups and the blank control group. Significance level: n.s., no significant difference; **p<0.01; ***p<0.001.

**Figure S5.** The effects of leucine and high glucose on neuronal cell viability. (a-d) The changes of cell viabilities in SH-SY5Y cells cultured with normal glucose (NG, 5.5 mM), high glucose (HG, 33 mM), leucine (LEU, 10 mM), and high glucose plus leucine (HG+LEU) for 6 h (a), 12 h (b), 24 h (c), and 36 h (d) (n = 5). Data were presented as means ± standard errors. The statistic difference between two groups was assessed by two-tailed unpaired student’s *t* test and *p*<0.05 was considered as a statistically significant difference. Pairwise comparisons were conducted between BCAAs treatment groups and the blank control group. Significance level: *p<0.05; ***p<0.001.

**Figure S6.** Excessive leucine aggravates neuronal injury and cognitive decline in diabetic mice. (a) Experimental procedure: In this study, type 1 diabetic (T1D) mice were established by intraperitoneal injection of streptozotocin (STZ) and treated with leucine for 12 weeks. Subsequently, learning and memory abilities of mice were evaluated by using the Morris water maze (MWM) test. (b) The escape latency during a 4-day training period of the MWM test in normal control (CON) mice, T1D mice, and T1D mice treated with leucine for 12 weeks (T1DL) (n = 8). (c) The swimming trajectories of CON, T1D and T1DL mice during the test period of the MWM test. (d-f) The platform frequence (d) and the percentages of swimming time (e) and distance (f) in the original platform area in CON, T1D and T1DL mice during the test period of the MWM test (n = 8). (g-j) Representative NeuN staining images (g) and the corresponding quantitative data in the CA1 (h), CA3 (i) and DG (j) regions of the hippocampus in T1D and T1DL mice (n = 6-7). Scale bar = 100 μm. (k) The mRNA expression levels of synaptic plasticity-related proteins in the hippocampus of T1D and T1DL mice (n = 6). The statistic difference among different groups was analyzed by one-way ANOVA with a post-hoc Dunnett’s test for multiple comparisons, and different lowercase letters indicate statistically significant differences among groups (*p*<0.05). The statistic difference between two groups was assessed by two-tailed unpaired student’s *t* test and *p*<0.05 was considered as a statistically significant difference. Significance level: *p<0.05; **p<0.01; ***p<0.001. The significant of difference in the escape latency during a 4-day training period was evaluated by two-way repeated measure ANOVA: G*T, interaction effect of group and time; G, main effect of group; T, main effect of time.

**Figure S7.** Exogenous leucine supplementation exacerbates neuronal injury in the hippocampus of diabetic mice. (a-d) Representative images of H&E (a, c) and Nissl (b, d) staining in the CA1, CA3 and DG regions of the hippocampus in type 1 diabetic (T1D) mice and T1D mice treated with leucine (T1DL) for 8 and 12 weeks. Scale bar = 100 μm.

**Figure S8.** Intermittent leucine deprivation (ILD) mitigates neuronal injury in the hippocampus of diabetic mice. (a, b) Representative images of H&E (a) and Nissl (b) staining in the CA1, CA3 and DG regions of the hippocampus in type 1 diabetic (T1D) mice and T1D mice treated with ILD for 12 weeks. Scale bar = 100 μm.

**Figure S9.** Changes of BCAAs catabolic enzymes in type 1 diabetic mice. (a-d) The mRNA expression levels of branched-chain ketoacid dehydrogenase (BCKDH) and branched-chain aminotransferase (BCAT) in the liver (a, b) and muscle (c, d) of normal control (CON) and type 1 diabetic (T1D) mice by RT-qPCR (n = 6). Data were presented as means ± standard errors. The statistic difference between two groups was assessed by two-tailed unpaired student’s *t* test and *p*<0.05 was considered as a statistically significant difference. Significance level: *p<0.05; **p<0.01.

**Figure S10.** *Akkermansia muciniphila* reduces neuronal injury in the hippocampus of diabetic mice. (a, b) Representative images of H&E (a) and Nissl (b) staining in the CA1, CA3 and DG regions of the hippocampus in type 1 diabetic (T1D) mice and T1D mice treated with *A. muciniphila* (AKK). Scale bar = 100 μm.

**Figure S11.** Excessive leucine accelerates neuronal injury and cognitive decline in antibiotic-treated mice. (a) Experimental procedure: Mice were treated with a broad-spectrum antibiotic (ABX) cocktail to deplete the endogenous gut bacteria and administered leucine by oral gavage for 2 weeks, followed by the MWM test. (b-d) The plate-counting experiments showing the levels of aerobic and anaerobic bacteria in the intestinal contents of control (CON) and ABX-treated mice as well as mice treated with antibiotics plus leucine (ABXL). (e-g) The concentrations of leucine in the intestinal contents (e), serum (f) and hippocampus (g) of CON, ABX and ABXL mice by LC-MS analysis (n = 6). (h) The escape latency during a 4-day training period of the MWM test in CON, ABX and ABXL mice (n = 6). (i) The swimming trajectories of CON, ABX and ABXL mice during the test period of the MWM test. (j-l) The percentages of swimming time (j) and distance (k) in the original platform area and the platform frequence (l) in CON, ABX and ABXL mice during the test period of the MWM test (n = 6). (m-p) Representative NeuN staining images (m) and the corresponding quantitative data in the CA1 (n), CA3 (o) and DG (p) regions of the hippocampus in CON, ABX and ABXL mice (n = 6). Scale bar = 100 μm. (q-u) The mRNA expression levels of synaptic plasticity-related proteins including SYP (q), PSD95 (r), EGR-1 (s), c-FOS (t) and ARC (u) in the hippocampus of CON, ABX and ABXL mice by RT-qPCR (n = 6). The statistic difference among different groups was analyzed by one-way ANOVA with a post-hoc Dunnett’s test for multiple comparisons, and different lowercase letters indicate statistically significant differences among groups (*p*<0.05). The significant of difference in the escape latency during a 4-day training period was evaluated by two-way repeated measure ANOVA: G*T, interaction effect of group and time; G, main effect of group; T, main effect of time.

**Figure S12.** Excessive leucine promotes neuronal injury in the hippocampus of antibiotic-treated mice. (a, b) Representative images of H&E (a) and Nissl (b) staining in the CA1, CA3 and DG regions of the hippocampus in control (CON) and ABX-treated mice as well as mice treated with antibiotics plus leucine (ABXL). Scale bar = 100 μm.

**Figure S13.** Changes in key enrichment pathways in SH-SY5Y cells treated with high glucose and leucine. (a) Commonly altered enrichment pathways in SH-SY5Y cells treated with high glucose (HG, 33 mM) and HG plus leucine (HGL) compared with normal glucose (NG, 5.5 mM). (b) Commonly altered enrichment pathways in SH-SY5Y cells treated with HGL compared with NG and HG.

**Figure S14.** The effects of high glucose and leucine on apoptosis in SH-SY5Y cells. (a, b) Enrichment plots of apoptosis in SH-SY5Y cells treated with high glucose (HG, 33 mM) and HG plus leucine (HGL) relative to normal glucose (NG, 5.5 mM).

**Figure S15.** The effect of leucine on ROS production in SH-SY5Y cells. Representative DCF immunofluorescence staining images in SH-SY5Y cells treated with high glucose (HG, 33 mM) and HG plus leucine (HGL), and the corresponding quantitative data (n = 6). Scale bar = 50 μm. Data were presented as means ± standard errors. The statistic difference between two groups was assessed by two-tailed unpaired student’s *t* test and *p*<0.05 was considered as a statistically significant difference. Significance level: ***p<0.001.

**Figure S16.** The effect of leucine on mitochondrial membrane potential and ROS production in primary neurons. (a) Representative JC-1 immunofluorescence staining images in primary neurons treated with high glucose (HG) and HG plus leucine (HGL), and the ratio of aggregate to monomer fluorescence (n = 3). (b) Representative DCF immunofluorescence staining images in primary neurons treated with HG and HGL, and the corresponding quantitative data (n = 3). Scale bar = 50 μm. Data were presented as means ± standard errors. The statistic difference between two groups was assessed by two-tailed unpaired student’s *t* test and *p*<0.05 was considered as a statistically significant difference. Significance level: **p<0.01.

**Figure S17.** Identification of TBK1 protein by LC-MS/MS. The MS/MS spectrum of peptides in TBK1 protein was acquired by using liquid chromatography coupled to tandem mass spectrometry (LC-MS/MS).

**Figure S18.** Leucine promotes mitophagy in SH-SY5Y cells by TBK1-OPTN axis. (a) Co-immunoprecipitation (Co-IP) experiments showing an increased level of the serine phosphorylation (p-Ser) of OPTN and an enhanced interactions of OPTN with TBK1, ubiquitin (Ub) and LC3B in SH-SY5Y cells. (b) Representative immunofluorescence staining images on the colocalization of OPTN with TBK1, LC3B and TOM20 in SH-SY5Y cells. Scale bar = 5 μm.

**Figure S19.** TBK1 knockdown protects against leucine-induced mitochondrial injury in the hippocampus of diabetic mice. (a-c) Representative TOM20 staining images and the corresponding quantitative data in the CA1 (a), CA3 (b) and DG (c) regions of the hippocampus in type 1 diabetic (T1D) mice treated with empty vector (shNC+T1D) and shTBK1 (shTBK1+T1D) as well as leucine-fed T1D (T1DL) mice treated with empty vector (shNC+T1DL) and shTBK1 (shTBK1+T1DL) (n = 7-8). Scale bar = 100 μm. Data were presented as means ± standard errors. The statistic difference with multiple groups was performed by two-way ANOVA followed by Sidak post hoc test. Significance level: n.s., no significant difference; *p<0.05; **p<0.01; ***p<0.001.

**Figure S20.** TBK1 knockdown alleviates leucine-induced neuronal injury in the hippocampus of diabetic mice. (a, b) Representative images of H&E (a) and Nissl (b) staining in the CA1, CA3 and DG regions of the hippocampus in type 1 diabetic (T1D) mice treated with empty vector (shNC+T1D) and shTBK1 (shTBK1+T1D) as well as leucine-fed T1D (T1DL) mice treated with empty vector (shNC+T1DL) and shTBK1 (shTBK1+T1DL). Scale bar = 100 μm.

**Figure S21.** TBK1 knockdown improves leucine-induced memory impairment in diabetic mice. Changes in the swimming trajectories during the test period of the Morris water maze test (MWM) in type 1 diabetic (T1D) mice treated with empty vector (shNC+T1D) and shTBK1 (shTBK1+T1D) as well as leucine-fed T1D (T1DL) mice treated with empty vector (shNC+T1DL) and shTBK1 (shTBK1+T1DL).

**Figure S22.** TBK1 knockdown reduces leucine-induced mitochondrial injury in SH-SY5Y cells under a high glucose condition. (a) Relative mRNA level of TBK1 in SH-SY5Y cells treated with three different siRNA sequences by RT-qPCR (n = 3). (b, c) The protein level of TBK1 analyzed by Western blotting (b) and the corresponding quantitative data (c) in SH-SY5Y cells treated with three different siRNA sequences (n = 4). (d) Representative immunofluorescence staining images on the colocalization of TOM20 and LC3B in SH-SY5Y cells treated with empty vector (si-NC) and si-TBK1 under high glucose (HG) and HG plus leucine (HGL) conditions. Scale bar = 5 μm. (e) Representative JC-1 immunofluorescence staining images and the ratio of aggregate to monomer fluorescence in SH-SY5Y cells treated with si-NC and si-TBK1 under HG and HGL conditions (n = 6). Scale bar = 50 μm. (f) The mRNA expression levels of mitochondrial function-related proteins in SH-SY5Y cells treated with si-NC and si-TBK1 under HG and HGL conditions by RT-qPCR (n = 5). (g) Representative DCF immunofluorescence staining images in SH-SY5Y cells treated with si-NC and si-TBK1 under HG and HGL conditions, and the corresponding quantitative data (n = 6). Scale bar = 50 μm. Data were presented as means ± standard errors. The statistic difference between two groups was assessed by two-tailed unpaired student’s *t* test and *p*<0.05 was considered as a statistically significant difference. The statistic difference with multiple groups was performed by two-way ANOVA followed by Sidak post hoc test. Significance level: n.s., no significant difference; **p<0.01; ***p<0.001.

**Figure S23.** Changes of BCAAs catabolic enzymes in type 2 diabetic mice. (a-d) The mRNA expression levels of branched-chain ketoacid dehydrogenase (BCKDH) and branched-chain aminotransferase (BCAT) in the liver (a, c) and muscle (b, d) of normal control (CON) and type 2 diabetic (T2D) mice by RT-qPCR (n = 8-9). Data were presented as means ± standard errors. The statistic difference between two groups was assessed by two-tailed unpaired student’s *t* test and *p*<0.05 was considered as a statistically significant difference. Significance level: n.s., no significant difference.

**Figure S24.** Neuronal injury occurs in the hippocampus of type 2 diabetic mice. (a, b) Representative images of H&E (a) and Nissl (b) staining in the CA1, CA3 and DG regions of the hippocampus in normal control (CON) and type 2 diabetic (T2D) mice. Scale bar = 100 μm.
